# ToxR Antagonizes H-NS Regulation of Horizontally Acquired Genes to Drive Host Colonization

**DOI:** 10.1371/journal.ppat.1005570

**Published:** 2016-04-12

**Authors:** Misha I. Kazi, Aaron R. Conrado, Alexandra R. Mey, Shelley M. Payne, Bryan W. Davies

**Affiliations:** 1 Department of Molecular Biosciences, The University of Texas at Austin, Austin, Texas, United States of America; 2 Institute of Cellular and Molecular Biology, The University of Texas at Austin, Austin, Texas, United States of America; Northwestern University, Feinberg School of Medicine, UNITED STATES

## Abstract

The virulence regulator ToxR initiates and coordinates gene expression needed by *Vibrio cholerae* to colonize the small intestine and cause disease. Despite its prominence in *V*. *cholerae* virulence, our understanding of the direct ToxR regulon is limited to four genes: *toxT*, *ompT*, *ompU* and *ctxA*. Here, we determine ToxR’s genome-wide DNA-binding profile and demonstrate that ToxR is a global regulator of both progenitor genome-encoded genes and horizontally acquired islands that encode *V*. *cholerae’*s major virulence factors and define pandemic lineages. We show that ToxR shares more than a third of its regulon with the histone-like nucleoid structuring protein H-NS, and antagonizes H-NS binding at shared binding locations. Importantly, we demonstrate that this regulatory interaction is the critical function of ToxR in *V*. *cholerae* colonization and biofilm formation. In the absence of H-NS, ToxR is no longer required for *V*. *cholerae* to colonize the infant mouse intestine or for robust biofilm formation. We further illustrate a dramatic difference in regulatory scope between ToxR and other prominent virulence regulators, despite similar predicted requirements for DNA binding. Our results suggest that factors in addition to primary DNA structure influence the ability of ToxR to recognize its target promoters.

## Author Summary

The transcription factor ToxR initiates a virulence regulatory cascade required for *V*. *cholerae* to express essential host colonization factors and cause disease. Genome-wide expression studies suggest that ToxR regulates many genes important for *V*. *cholerae* pathogenesis, yet our knowledge of the direct regulon controlled by ToxR is limited to just four genes. Here, we determine ToxR’s genome-wide DNA-binding profile and show that ToxR is a global regulator of both progenitor genome-encoded genes and horizontally acquired islands that encode *V*. *cholerae’*s major virulence factors. Our results suggest that ToxR has gained regulatory control over important acquired elements that not only drive *V*. *cholerae* pathogenesis, but also define the major transitions of *V*. *cholerae* pandemic lineages. We demonstrate that ToxR shares more than a third of its regulon with the histone-like nucleoid structuring protein H-NS, and antagonizes H-NS for control of critical colonization functions. This regulatory interaction is the major role of ToxR in *V*. *cholerae* colonization, since deletion of *hns* abrogates the need for ToxR in *V*. *cholerae* host colonization. By comparing the genome-wide binding profiles of ToxR and other critical virulence regulators, we show that, despite similar predicted DNA binding requirements, ToxR is unique in its global control of progenitor-encoded and acquired genes. Our results suggest that factors in addition to primary DNA structure determine selection of ToxR binding sites.

## Introduction

Bacteria emerge as pathogens by horizontally acquiring new genetic functions from their environment and neighboring organisms [1,2]. *Vibrio cholerae*, the etiological agent of cholera, is a paradigm of this process. Benign environmental *V*. *cholerae* isolates emerge as pandemic pathogens through the horizontal acquisition and incorporation of genetic elements encoding virulence factors into their progenitor genomes [3–5]. The factors gained by the benign progenitor genome include cholera toxin, encoded on the CTX prophage, and the colonization pilus TCP, along with regulators TcpP and ToxT, encoded on the Vibrio Pathogenicity Island 1 (VPI-1) [6–9]. Moreover, current 7^th^ pandemic *V*. *cholerae* strains are genetically distinguished from the previous 6^th^ pandemic strains by the acquisition of two new horizontally acquired elements, Vibrio Seventh Pandemic islands 1 and 2 (VSP-1, 2) [5,10]. The acquisition of VSP-1 and 2 are thought to have promoted the emergence and dominance of 7^th^ pandemic strains.

The progenitor genome-encoded transcription factor ToxR plays a critical role in *V*. *cholerae* virulence and stress response. ToxR is a membrane-bound transcriptional regulator with a partner protein, ToxS, that enhances ToxR activity [4,11,12]. The major role of ToxR in pathogenesis is to act with TcpP and induce expression of *toxT*. ToxT then triggers expression of genes encoding colonization factors and cholera toxin, resulting in disease [13–17]. When overexpressed, or in the presence of bile, ToxR can also directly activate the genes encoding cholera toxin, *ctxAB* [18,19]. On the progenitor genome, ToxR directly regulates expression of *V*. *cholerae*’s major outer membrane proteins: OmpU and OmpT [20,21]. Expression of OmpU and OmpT is important for *V*. *cholerae* to survive host-relevant stresses including bile, antimicrobial peptides, and pH changes [22–25]. ToxR’s ability to regulate both progenitor-encoded and recently acquired DNA allows for new and existing gene functions to be coordinated, which has supported *V*. *cholerae’s* emergence as a successful pathogen.

ToxR expression and activity are responsive to stimuli, including pH, oxygen, temperature, and metabolites [24,26–28]. Other transcription factors likely compete with ToxR for binding sites to control gene expression under different conditions [29–31]. The complexity of ToxR regulation may be necessitated by the many processes ToxR impacts [32,33]. Despite its critical role in virulence, ToxR has only been shown to directly regulate four target genes [15,20,34,35]. Here, we integrate chromatin-immunoprecipitation sequencing (ChIP-seq) data with gene expression data and phenotype studies to map the regulon directly controlled by ToxR. We identify ToxR regulation in several new roles affecting *V*. *cholerae* virulence and biofilm formation, which correlate with the emergence of 7^th^ pandemic strains. Analysis of our ChIP data was unable to identify a motif that could explain how ToxR identified its target binding location *in vivo*. However, it did describe an affinity of ToxR for low GC-content locations that were frequently shared with the histone-like nucleoid structuring protein H-NS (VC1130;VicH). Our results show ToxR antagonizes H-NS transcriptional regulation, and that this interplay controls *V*. *cholerae* host colonization and impacts biofilm formation. A comparison between ToxR and additional prominent virulence regulators TcpP and ToxT shows a unique global role for ToxR gene regulation.

## Results

### Characterization of the ToxR (VC0984) regulon

ToxR is a major virulence regulator in *V*. *cholerae*, yet we only know of four genes that it can directly regulate: *toxT*, *ompU*, *ompT* and *ctxA* [13,15,19–21,36]. Microarray experiments performed under conditions that induce virulence factor expression have implicated ToxR in the regulation of more than 100 genes [33], suggesting a much larger regulon. However, it is unclear how much of this regulation is direct. To determine the direct regulon of ToxR, we used chromatin-immunoprecipitation-sequencing (ChIP-seq) to identify ToxR binding sites across the genome. We ectopically expressed ToxR with a C-terminal V5 tag under control of an arabinose inducible promoter in 7^th^ pandemic *V*. *cholerae* strain C6706. This approach allows reproducible induction and immunoprecipitation of ToxR without prior knowledge of all the environmental factors that may control its expression. Expression levels of ToxR are shown S1A Fig. This method has proven effective for ChIP-seq in *V*. *cholerae* and other bacteria [37–40].

To confirm the DNA binding activity of the tagged ToxR, we induced its expression and performed ChIP as previously described [37,38]. Quantitative PCR (qPCR) analysis of ToxR ChIP DNA samples demonstrates that V5-tagged ToxR strongly binds known target sites in the *toxT*, *ompT* and *ompU* promoters, but not to a negative control site at the *icd* promoter (S2 Fig). We performed ChIP-seq and identified genome-wide ToxR binding locations as previously described [37,38]. Alignment of sequencing reads from each sample gave average genome coverage of 41-fold. This depth of coverage allowed us to use a stringent false-discovery rate (FDR) cutoff of 0.001% to identify ToxR ChIP-enriched genomic regions, which are referred to as peaks. ChIP peaks are identified when the sequence coverage of a given genomic region in the experimental sample exceeds the non-immunoprecipitated input control sample at a rate specified by the FDR. ChIP peak enrichment ranged from 5—to 19-fold over the input. qPCR analysis of ChIP DNA generally showed a much higher fold enrichment (S2 Fig). This is likely because computational ChIP-seq enrichment is a measurement of the average enrichment across the whole peak, while our qPCR analysis generally measures enrichment at specific locations within the peak.

We compared the ToxR ChIP peak lists generated from two biological replicates and set a limit that a peak must be identified in both replicates to be included as a potential ToxR binding location for our analysis. Peaks meeting this standard were then manually curated for accuracy [41]. We associated a ToxR peak with a gene based on its proximity to promoters and translation start sites. With these criteria, a ToxR peak can associate with more than one gene if 1) the translational start sites of two or more genes are close together, or 2) if ToxR binds multiple sites that are too close together to be accurately separated by peak-calling algorithms [42]. In these cases we used published gene expression data and data generated in this study to interpret which gene(s) ToxR is likely to directly regulate. For example, there is a ToxR peak overlapping the 172 bases between divergently transcribed genes VC0844 and VC0845. Previous studies have described ToxR affecting regulation of both genes [33,43].

Our analysis identified 35 ToxR peaks associated with 39 genes by our criteria (Table 1). Three ToxR peaks remained associated with more than one gene. The coordinates encompassing the raw ToxR ChIP-seq peak locations and their associated genes are given in S1 Table. Schematics of ToxR ChIP enrichment at select loci are shown in S3 Fig. One peak was identified covering each of the promoters for *toxT*, *ompU*, and *ompT*, validating our procedure for identifying ToxR binding locations. Table 1 shows several genes in horizontally acquired elements and genes that have previously been connected with ToxR regulation through microarray and additional studies. Analysis of the locations and functions of genes associated with ToxR peaks identified two overrepresented groups: 18% of the genes identified in this study are known or predicted to function in biofilm formation, and 40% are located on horizontally acquired elements.

**Table 1:** Genes associated with ToxR ChIP-seq peaks.

Gene	Function	Genomic Island	ToxR Regulation^a^	H-NS Associated
*ryhB*	Iron-regulated sRNA	-	-	-
VC0176	Transcriptional regulator	VSP-I	Yes (33)	Yes
VC0177	Hypothetical protein	VSP-I	-	Yes
VC0178	Patatin-like protein	VSP-I	-	-
VC0182	Hypothetical protein	VSP-I	-	Yes
VC0183	Hypothetical protein	VSP-I	-	Yes
VC0260 (*galE*)	O-antigen biosynthesis	-	-	Yes
VC0269 (*manA*)	O-antigen biosynthesis	-	-	Yes
VC0280 (*cadB*)	Lysine/cadaverine antiporter	-	-	-
VC0423	Arginine deiminase	-	Yes (33)	-
VC0490	Hypothetical protein	VSP-II	Yes (33)	Yes
VC0493	Hypothetical protein	VSP-II	Yes (33)	-
VC0501	pseudogene	VSP-II	-	-
VC0633 (*ompU*)	Outer membrane protein	-	Yes (20,21,33)	-
VC0824 (tagD)	Thiol peroxidase	VPI-1	Yes (33)	Yes
VC0825 (*tcpI*)	Toxin co-regulated pilus protein	VPI-1	-	Yes
VC0838 (*toxT*)	Virulence transcriptional regulator	VPI-1	Yes (13,36)	Yes
VC0844 (*acfA*)	Accessory colonization factor	VPI-1	Yes (33)	Yes
VC0845 (*acfD*)	Accessory colonization factor	VPI-1	Yes (33,43)	Yes
VC0880	putative transporter	-	-	-
VC0934 (*vpsL*)	Capsular polysaccharide biosynthesis	-	-	Yes
VC0972	Chitin transporter	-	Yes (33)	-
VC0988 (*tppB*)	Tripeptide transporter permease	-	-	-
VC1145	Hypothetical protein	-	-	-
VC1197	Hypothetical protein	-	Yes (33)	-
VC1330	Hypothetical protein	-	-	Yes
VC1398 (*cheY*)	Chemotaxis protein	-	-	-
VC1599	GGDEF family protein	-	-	-
VC1613	Hypothetical protein	-	-	-
VC1649	Serine protease	-	Yes (33)	-
VC1762	Hypothetical protein	VPI-2	-	-
VC1773	Hypothetical protein	VPI-2	-	Yes
VC1800	Hypothetical protein	VPI-2	-	-
VC1839	TolQ protein	-	-	-
VC1854 (*ompT*)	Outer membrane protein	-	Yes (20,21,33)	-
VC1856	Hypothetical protein	-	-	-
VC2013	phosphate transport system	-	Yes (33)	-
VC2485 (*leuO*)	Leucine transcriptional activator	-	Yes (33,51)	-
VC2697	GGDEF family protein	-	-	-

^a^ select citations for previously reported ToxR regulation are shown in brackets.

### ToxR positively and negatively regulates genes involved in biofilm formation

We identified ToxR peaks in the promoter regions of six genes and one small RNA (sRNA) all known or suspected to play a role in biofilm formation: *ryhB*, *vpsL* (VC0934), VC1145, VC1330, VC1599, *leuO* (VC2485), and VC2697 [44–49]. These genes are all encoded on the progenitor genome [50]. ToxR was previously shown to induce *leuO* expression [51]. Our ChIP-seq analysis identified ToxR binding covering the *leuO* promoter region (Table 1), which shows that the observed positive regulation is likely direct. To further understand how ToxR regulates expression of genes involved in biofilm formation, we determined the impact of ToxR on the expression of *ryhB*, *vpsL*, and VC1599. These genes were chosen because they have not been previously associated with ToxR regulation and encode diverse biological functions. RyhB is a small regulatory RNA involved in regulation of iron metabolism [46,52]. VC1599 is a diguanylate cyclase that produces the signaling molecule cyclic-di-GMP (cdiGMP) [45,53]. *vpsL* encodes a glycosyltransferase for *Vibrio* polysaccharide production and is the first gene of the *Vibrio* polysaccharide *vps*-II operon [44,54,55].

**Fig 1:**
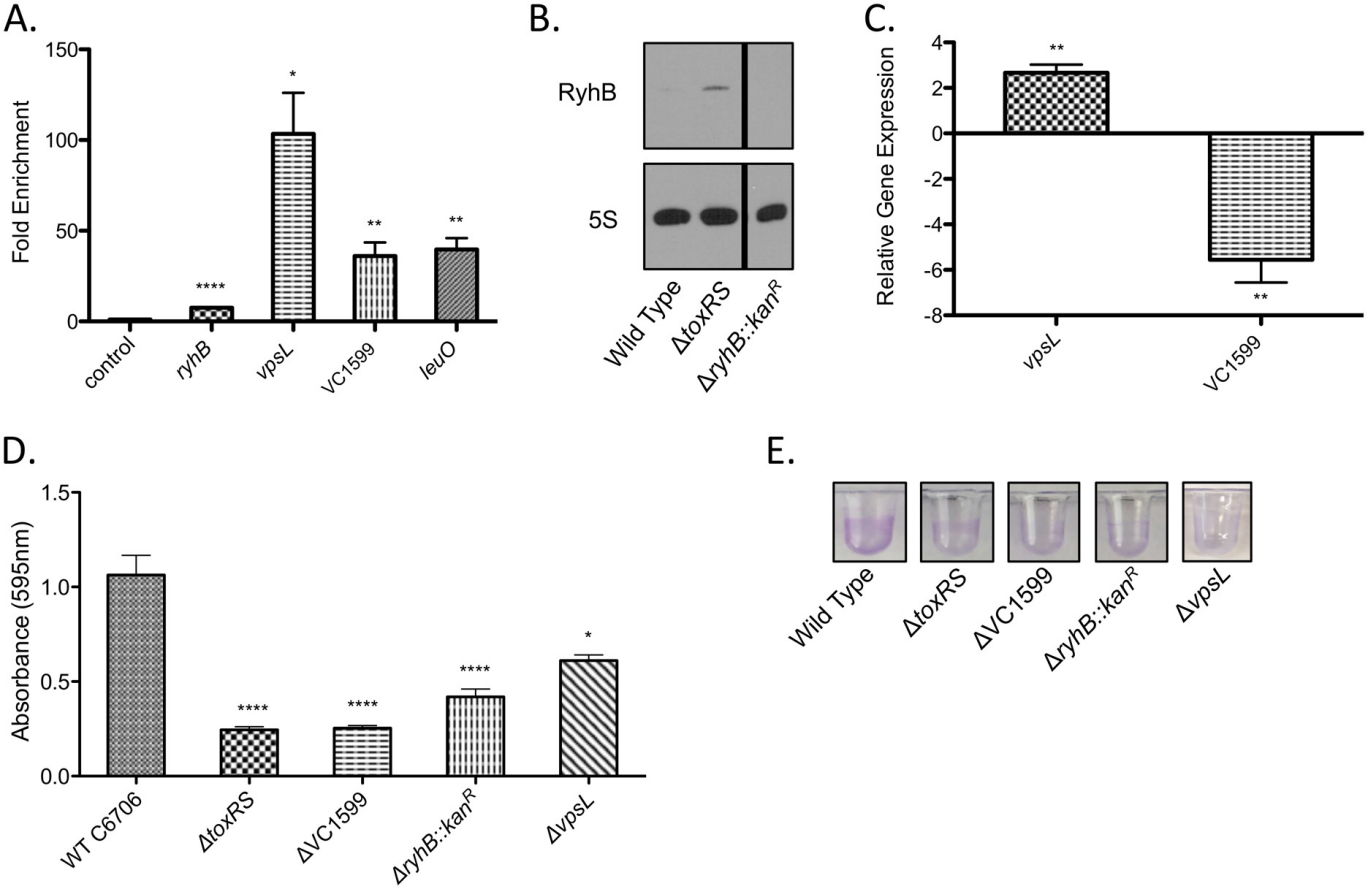
ToxR positively and negatively regulates genes important for biofilm formation. (A) ToxR ChIP fold enrichment of the promoter regions of *ryhB*, *vpsL*, VC1599, and *leuO*. Enrichment of a non-ToxR regulated *icd* promoter region is shown as a negative control. ToxR enrichment of the *ryhB*, *vpsL*, and VC1599 promoter regions is statistically significant relative to the *icd* promoter. ****p < 0.0001; **p < 0.01; *p < 0.05, unpaired two-tailed Student’s *t* test. (B) Northern blot for RyhB. Equal amounts of total RNA were loaded. The 5S blot is shown for a loading control. All Northern blots were performed in biological triplicate. RyhB expression increased 3.3 ± 0.1 fold in the Δ*toxRS* mutant compared to the wild type strain. Mean with standard error of the mean (SEM) reported, p < 0.001, unpaired two-tailed Student’s *t* test. A representative image is shown. All samples for this image were processed on the same gel. (C) qRT-PCR analysis of *vpsL* and VC1599 gene expression. The expression level of these genes in the Δ*toxRS*+p*toxRS* strain is shown, normalized to expression levels in the Δ*toxRS*+vector control strain, which was set at 1. Expression of *vpsL* increased, while expression of VC1599 decreased in the Δ*toxRS*+p*toxRS* strain compared to the control. **p < 0.005, unpaired two-tailed Student’s *t* test. (D) Quantification of biofilm formation in rich broth at 30°C. Δ*toxRS*, ΔVC1599, Δ*ryhB*::*kan* ^*R*^ and Δ*vpsL* mutant strains show a defect in biofilm formation compared to the wild-type strain. ****p < 0.0001; *p < 0.05, unpaired two-tailed Student’s *t* test. (E) Representative images of biofilm formation. For panels A, C and D, mean with standard error of the mean (SEM) is shown.

qPCR analysis of ToxR ChIP DNA confirmed our sequencing data and showed ToxR enrichment of *ryhB*, *vpsL*, and VC1599 promoter regions, but not of a negative control site (Fig 1A). We used northern blots and quantitative reverse-transcription PCR (qRT-PCR) to determine ToxR regulation of *ryhB*, *vpsL* and VC1599. Northern blot analysis showed that deletion of *toxRS* led to an increase in *ryhB* abundance, consistent with direct ToxR repression of *ryhB* expression (Fig 1B). Deletion of *toxRS* alone did not affect *vpsL* or VC1599 expression (S4 Fig). The free-living planktonic cells used for our gene expression assays might not recapitulate the environmental signals needed for ToxR regulation of *vpsL* and VC1599 utilized for biofilm formation [56]. In an attempt to bypass this potential signaling hurdle, we compared expression of *vpsL* and VC1599 in a *toxRS* deletion strain carrying either an empty vector or a vector with an arabinose inducible *toxRS* operon to specifically increase ToxRS levels. In this comparison, induction of *toxRS* led to an increase in *vpsL* expression and a decrease in VC1599 expression, supporting direct ToxR regulation of these genes (Fig 1C). Our results establish both positive and negative control of biofilm-associated genes by ToxR. It also ties ToxR regulation to small regulatory RNAs and cdiGMP, both of which influence a wide spectrum of genes and biological processes [46,57] that may be responsible for indirect effects associated with ToxR regulation.

We assessed the ability of wild type, *toxRS*, *ryhB*, VC1599, and *vpsL* mutant strains to form biofilm in a static microtiter assay in rich broth at 30°C (Fig 1D and 1E). The *toxRS* deletion strain showed reduced biofilm formation, supporting its regulatory role in this process. This phenotype was complemented by ectopic expression of *toxRS* (S5 Fig). The requirement of *ryhB*, *vpsL*, and genes downstream of *vpsL* in the *vps*-II operon for biofilm formation was previously established [44,46,55,58]. Supporting those results, a *vpsL* in-frame deletion mutant and a Δ*ryhB*::*kan* ^*R*^ mutant both showed a defect in biofilm formation (Fig 1D and 1E). These phenotypes were complemented by ectopic expression of the respective gene (S5 Fig). Overexpression of VC1599 had been shown to increase biofilm formation [45]. Supporting this observation our VC1599 deletion strain showed decreased biofilm production (Fig 1D and 1E). This phenotype was complemented by ectopic expression of VC1599 from a plasmid, which led to biofilm overproduction (S5 Fig).

Loss of *toxRS* or *vpsL* decreased biofilm formation under the experimental conditions used for our assay. Positive regulation of *vpsL* by ToxR could explain the biofilm defect of our Δ*toxRS* mutant. We tested the ability of a Δ*toxRS*Δ*vpsL* double mutant to form biofilm, as well as Δ*toxRS*Δ*ryhB*::*kan* ^*R*^ and Δ*toxRS*ΔVC1599 double mutants. We did not observe a significant difference in biofilm formation for any double mutant relative to the Δ*toxRS* mutant (S6 Fig). The resolution of our assay may not be sufficient to identify synergies or additive effects of these mutants.

### ToxR regulates gene expression on all four *Vibrio* pathogenicity islands

**Fig 2:**
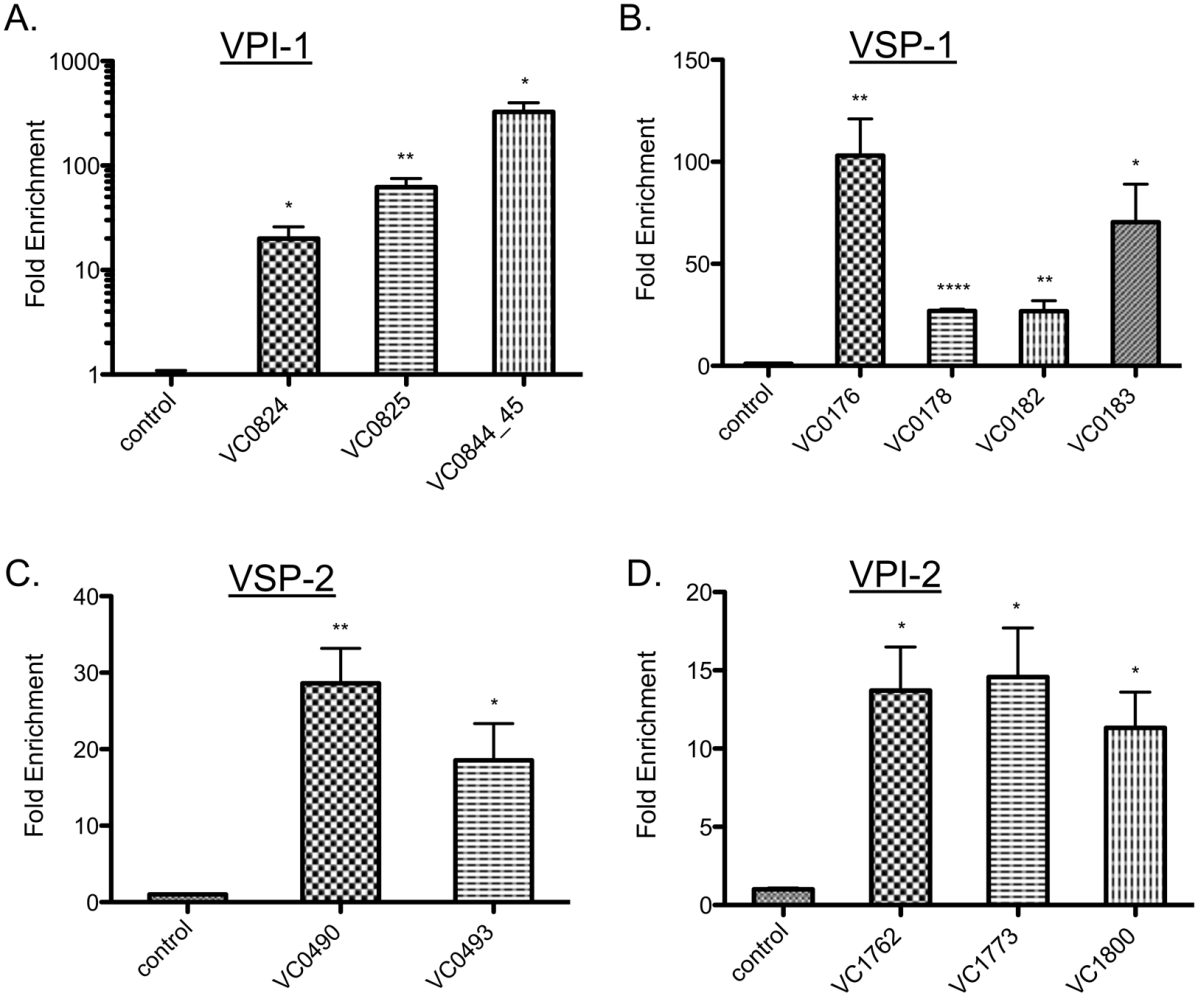
ToxR binds to promoter regions on all four major *Vibrio* pathogenicity islands. ToxR ChIP enrichment of the promoter regions of selected genes located on horizontally acquired islands (A) VPI-1, (B) VSP-1, (C) VSP-2, and (D) VPI-2. Enrichment of a non-ToxR-dependent promoter region of *icd* is shown as the negative control. ToxR enrichment of indicated promoter regions is statistically significant compared to the control. ****p < 0.0001; **p < 0.01; *p < 0.05, unpaired two-tailed Student’s *t* test. Mean with standard error of the mean (SEM) is shown.

Our ChIP-seq results showed that ToxR binds locations on all four of *V*. *cholerae*’s major acquired pathogenicity islands: VPI-1, VPI-2, VSP-1, and VSP-2 (Table 1). In addition to the *toxT* promoter, our analysis shows ToxR binds the promoter regions of VPI-1 genes VC0824 (*tagD*), VC0825 (*tcpI*), VC0844 (*acfA*), and VC0845 (*acfD*) (Table 1). qPCR analysis of ToxR ChIP DNA validated our sequencing results that ToxR binds the promoter regions of VC0824 (*tagD*), VC0825 (*tcpI*), and the promoter region shared by VC0844 (*acfA*) and VC0845 (*acfD*) (Fig 2A). Combined with gene expression studies describing positive regulation of *tagD*, *acfA*, and *acfD* genes by ToxR, independent of ToxT [33,43,59], our results support a direct role for ToxR in the positive regulation of these genes, expanding ToxR’s known targets on VPI-1. While the function of these genes is under investigation, *tcpI*, *acfA*, and *acfD* are known to be required for *V*. *cholerae* colonization of a model host [16,60].

When overexpressed or activated by specific compounds, ToxR can activate *ctxA* expression [18–20]. However, the physiological relevance of this interaction is unclear. Our ChIP-seq analysis did not identify a ToxR binding site in the *ctxA* promoter, suggesting the event was either below our level of detection or does not occur to an appreciable extent in *V*. *cholerae* under our experimental conditions.

**Fig 3:**
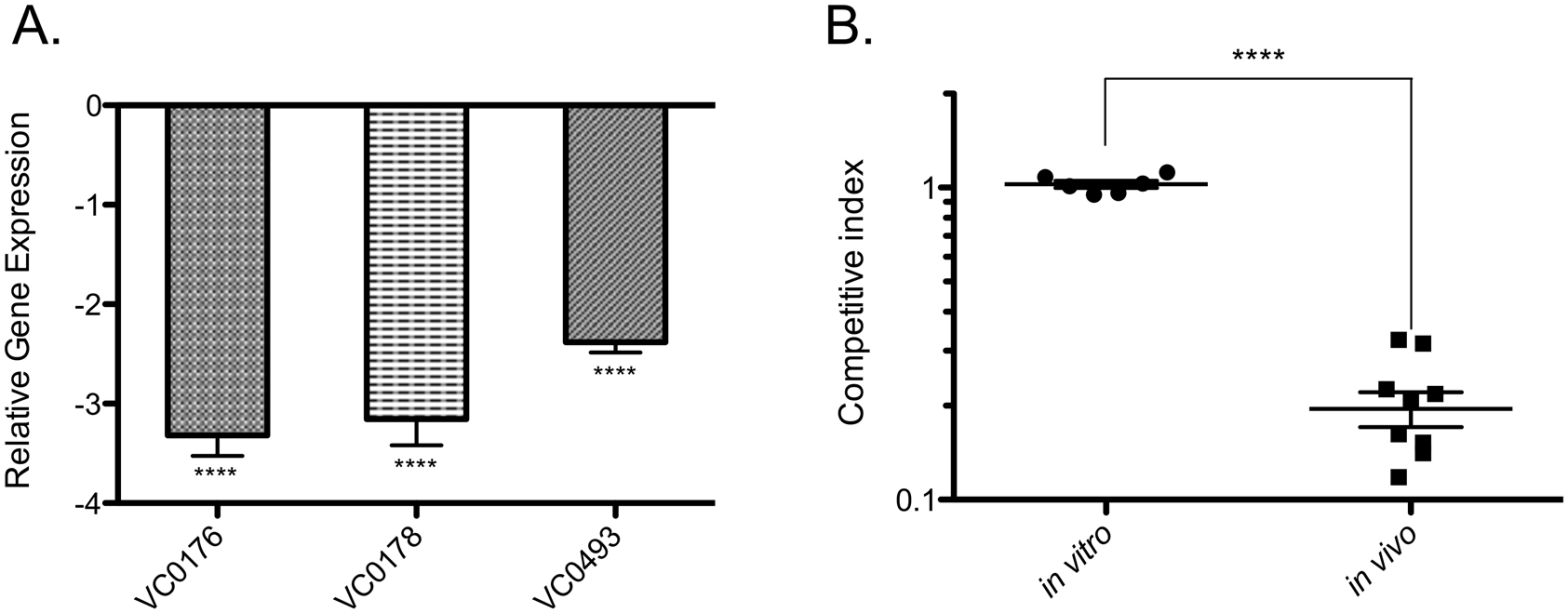
ToxR negatively regulates VSP-1 encoded genes affecting host colonization. (A) qRT-PCR analysis of VC0176, VC0178, and VC0493 gene expression. The expression level of these genes in the Δ*toxRS*+p*toxRS* strain is shown, normalized to expression levels in the Δ*toxRS*+vector control strain, which was set at 1. VC0176, VC0178, and VC0493 gene expression is decreased in Δ*toxRS*+p*toxRS* compared to the control. ****p < 0.0001, unpaired two-tailed Student’s *t* test. (B) Competition assays of ΔVC0176 mutant vs. wild type *in vitro* and *in vivo* in the infant mouse model. Each point represents an individual mouse result. ΔVC0176 has a colonization defect vs. wild type *in vivo* in the infant mouse model compared to *in vitro*. ****p < 0.0001, unpaired two-tailed Student’s *t* test.

Seventh pandemic *V*. *cholerae* is genetically distinguished from previous 6^th^ pandemic strains by the presence of acquired islands VSP-1 and 2. Little is known about the origin, content, and regulation of these islands, though VSP-1 carries at least one gene that influences the ability of *V*. *cholerae* to colonize the infant mouse model [38,61]. Our results show ToxR binding across the promoter regions of genes located on both VSP-1 and VSP-2 (Table 1). qPCR analysis of ToxR ChIP DNA validated that ToxR binds the promoter regions of VC0176, VC0178, VC0182, and VC0183 on VSP-1, and VC0490 and VC0493 on VSP-2 (Fig 2B and 2C). Microarray analysis suggested that ToxR can repress of VC0176, VC0490, and VC0493 expression under virulence-gene inducing conditions [33], supporting a direct role for ToxR in their regulation. To corroborate and expand ToxR regulation of VSP-1 and 2, we used qRT-PCR to determine if ToxR regulated expression of selected VSP-1 and 2 genes. Deletion of *toxRS* alone did not affect expression of VSP-1 or 2 genes when *V*. *cholerae* was grown exponentially in rich broth (S4 Fig). We again considered that conditions for ToxR regulation of VSP-1 and 2 genes were not recapitulated by exponentially growing cells in rich broth. We compared expression of VSP-1 and 2 genes in a *toxRS* deletion strain carrying an empty vector or a vector with an arabinose inducible *toxRS* operon. In this comparison, induction of *toxRS* led to repression of VC0176, VC0178, and VC0493, supporting a direct role for ToxR regulation of VSP-1 and VSP-2 genes [33] (Fig 3A).

Considering the central role of ToxR in virulence regulation, we questioned whether the ToxR-regulated genes on VSP-1 also affected *V*. *cholerae* colonization. VC0178 was previously shown not to influence *V*. *cholerae* host colonization; however, VC0176 was not tested [38]. We constructed an unmarked VC0176 deletion mutant and tested its ability to colonize the infant mouse. We found that the ΔVC0176 mutant showed approximately a 5-fold defect in colonizing the infant mouse intestine in competition with the parental strain (Fig 3B). No defect was observed when the strains were competed in liquid culture (Fig 3B). The phenotype was complemented by ectopic expression of VC0176 (S7 Fig).

These results expand the regulatory role of ToxR on virulence islands VPI-1 and 2, which are found in all pandemic *V*. *cholerae* strains. They further show that ToxR has gained control over expression of recently acquired genetic elements that define the current 7^th^ pandemic strains, including a new VSP-1 colonization factor VC0176. These results implicate ToxR as a regulatory hub for integrating expression of progenitor genome-encoded functions with newly acquired genes to promote *V*. *cholerae* fitness.

### ToxR antagonizes H-NS binding at shared locations

Our results demonstrate that ToxR binds all four *Vibrio* pathogenicity islands, and implicates ToxR as a global regulator of horizontally acquired genetic elements. Horizontally acquired DNA generally has a lower GC-content than the progenitor genome [62]. For example, the average GC-content of the N16961 *V*. *cholerae* genome is 47%, while the average GC-content of VPI-2 and VSP-1 is 42% and 40% respectively [63,64]. Analysis of the DNA sequences comprising the ToxR ChIP-seq peak locations showed they contain an average GC-content of just 40%. This suggests that ToxR preferentially binds DNA with base composition more similar to acquired elements than to that of the progenitor genome average. This result agrees with the low GC-content of the predicted ToxR consensus binding motif (TNAAA-N_5_-TNAAA), which was based on ToxR binding and/or activation of *toxT*, *ompT*, *ompU*, and *ctxA* promoters [15,20]. The preference for binding low GC-content DNA is shared with the histone-like nucleoid structuring protein (H-NS) that binds and silences horizontally acquired DNA [65]. *V*. *cholerae* H-NS binds and silences genes identified in our ToxR regulon study, including *toxT* and *vpsL* [31,66–68]. These observations prompted us to question if ToxR and H-NS may share additional genomic binding locations. We added a V5-tag to the C-terminus of the chromosomally encoded H-NS in *V*. *cholerae* C6706 to facilitate immunoprecipitation. We performed ChIP-seq for H-NS-V5 and determined its genome-wide binding profile under the same conditions as we used for ToxR ChIP-seq (S2 Table). We compared the genome binding profiles and found that 39% of regions bound by ToxR were also identified in our H-NS ChIP-seq analysis (Table 1).

**Fig 4:**
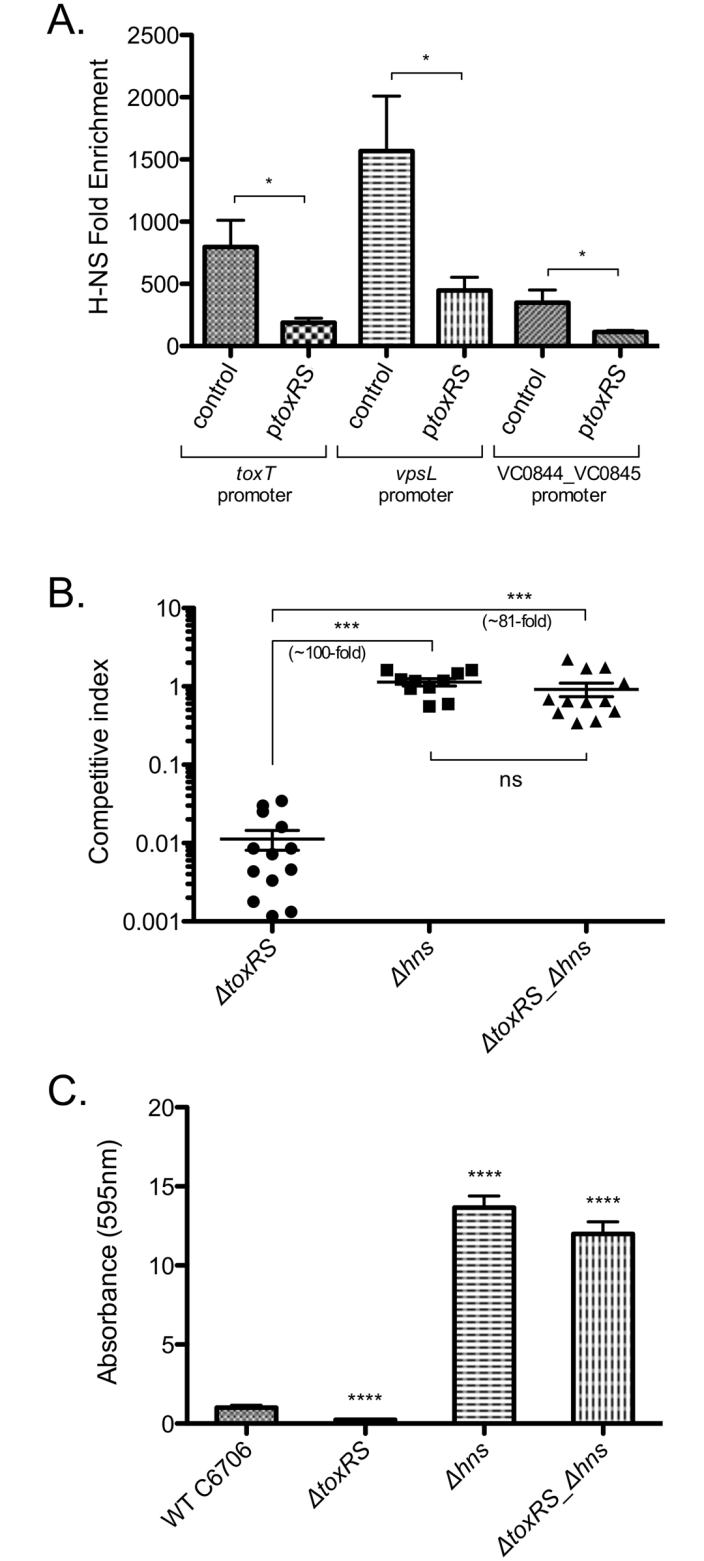
ToxR antagonizes H-NS function to control host colonization and biofilm formation. (A) ChIP enrichment of H-NS on the indicated promoter regions in the presence (p*toxRS*) or absence (control) of *toxRS* ectopic expression. H-NS enrichment on the indicated promoter regions is decreased in the presence of *toxRS* ectopic expression (p*toxRS*) compared to in the absence of *toxRS* ectopic expression (control). *p < 0.05, unpaired two-tailed Student’s *t* test. (B) Competition assays of indicated *V*. *cholerae* mutants vs. wild type strain in the infant mouse intestinal colonization model. The fold change difference between the indicated strains is shown alongside the statistical significance. Statistical significance was determined by One-Way ANOVA analysis followed by a Tukey’s multiple comparison post-test, ***p < 0.001. (C) Quantification of biofilm formation in rich broth at 30°C, All biofilm measurements were normalized to the wild-type strain which was set at 1. Δ*toxRS* mutant shows a defect in biofilm formation compared to the wild-type strain, while the Δ*hns* mutant and Δ*toxRS*Δ*hns* double mutant have increased biofilm formation compared to the wild-type strain. ****p < 0.0001, unpaired two-tailed Student’s *t* test. Mean with standard error of the mean (SEM) is shown.

Previous studies have shown genetic interactions between *toxR*, *tcpP*, and *hns* influence expression of the *toxT* promoter [31], and that H-NS can directly regulate *vpsL* [54,66]. Our results suggest that ToxR might antagonize H-NS regulation at multiple locations to gain access to gene targets. Rather than a defined consensus motif, topology has been implicated as a critical factor controlling H-NS binding to DNA. Low GC-content DNA forms structures that are preferentially bound by H-NS [69–71]. Since DNA topology and H-NS binding changes with environmental conditions [65,69,72,73] we wanted to test if ToxR could antagonize H-NS binding *in vivo*, in the context of the bacterial cell. To do this, we introduced an empty or arabinose-inducible, *toxRS-*encoding plasmid into our *V*. *cholerae* strain containing V5-tagged H-NS. We induced *toxRS* expression with arabinose and performed ChIP against H-NS-V5. We next used qPCR to determine H-NS enrichment at shared ToxR binding locations. We chose to examine the *vpsL* promoter on the progenitor genome, and *toxT* and VC0844-5 promoter regions on VPI-1. At each location we found that H-NS occupancy decreased following induction of *toxRS*, indicating that ToxR can antagonize H-NS binding at these locations (Fig 4A). These experiments were performed in the presence of the chromosomally-encoded *toxRS*. Thus, the impact of ToxR on H-NS binding may be even greater than observed here. As H-NS is a global silencer of horizontally acquired genetic material, our results indicate that ToxR has the ability to antagonize H-NS binding and bring the regulation of new genetic material under virulence gene control.

### The genetic interaction of ToxR with H-NS controls host colonization and biofilm formation

ToxR is essential for *V*. *cholerae* virulence through its regulation of many genes important for host colonization and pathogenesis [13–17]. Supporting this role, our Δ*toxRS* deletion strain was strongly outcompeted by the wild type strain in infant mouse intestinal colonization assays (Fig 4B), which agreed with previous reports [74]. This defect was complemented by ectopic expression of *toxRS* (S8 Fig). H-NS represses many virulence genes, and deletion of *hns* results in their induction [31], suggesting deletion of *hns* should not impair *V*. *cholerae* intestinal colonization. Supporting our hypothesis the Δ*hns* mutant did not show a significant defect in colonizing the infant mouse intestine in competition with the wild type strain (Fig 4B). Our data showed that ToxR and H-NS both bind the promoter regions of many of the same genes that are important for *V*. *cholerae* virulence (Table 1). It also showed that ToxR could antagonize H-NS binding at shared binding locations (Fig 4A). If ToxR antagonizes H-NS repression of important colonization factors, then deletion of H-NS should alleviate the need for ToxR regulation in intestinal colonization. To genetically test our hypothesis, we constructed a double Δ*toxRS*Δ*hns* mutant and assayed its ability to colonize infant mice (Fig 4B). Agreeing with our hypothesis for the importance of ToxR’s genetic interaction with H-NS for colonization, the double Δ*toxRS*Δ*hns* mutant showed no competitive defect compared to wild type or the Δ*hns* mutant alone. Ectopic expression of *hns* in the Δ*toxRS*Δ*hns* mutant produces a competition defect similar to that of *ΔtoxRS* mutant alone (S8 Fig). Removing H-NS activity genetically eliminates the need for ToxR regulation in *V*. *cholerae* host colonization.

We observed a similar genetic effect for biofilm formation, where deletion of *hns* compensated for the biofilm defect of the *toxRS* mutant (Fig 4C). This effect was complemented by ectopic expression of *hns*, though not to wild type levels (S9 Fig). This may be because expression of *hns* from a plasmid does not recapitulate H-NS levels necessary for normal biofilm regulation in our strain. Our results indicate that for both host colonization and biofilm formation, the major purpose of the ToxR regulation is to antagonize H-NS activity.

### ToxR does not partner with TcpP for global regulation

ToxR co-operates with transcription factor TcpP to activate *toxT* gene expression [13–17]. Like ToxR, TcpP is a membrane-bound transcription factor with an enhancer partner protein, TcpH, and is responsive to environmental conditions and upstream regulation [7,26,31,75,76]. TcpP is only known to regulate *toxT*. The region of the *toxT* promoter that affects TcpP binding also shows low GC-content and low sequence complexity (TGTAA-N_6_-TGTAA) [77]. Given the similarity of TcpP’s and ToxR’s binding motifs, we hypothesized that TcpP may also directly regulate more genes, alone or in association with ToxR. Previous microarray studies found that deletion of *tcpP* changed the expression of 58 genes under conditions that activate colonization factor expression [33], supporting a possible broader role for TcpP regulation.

**Fig 5:**
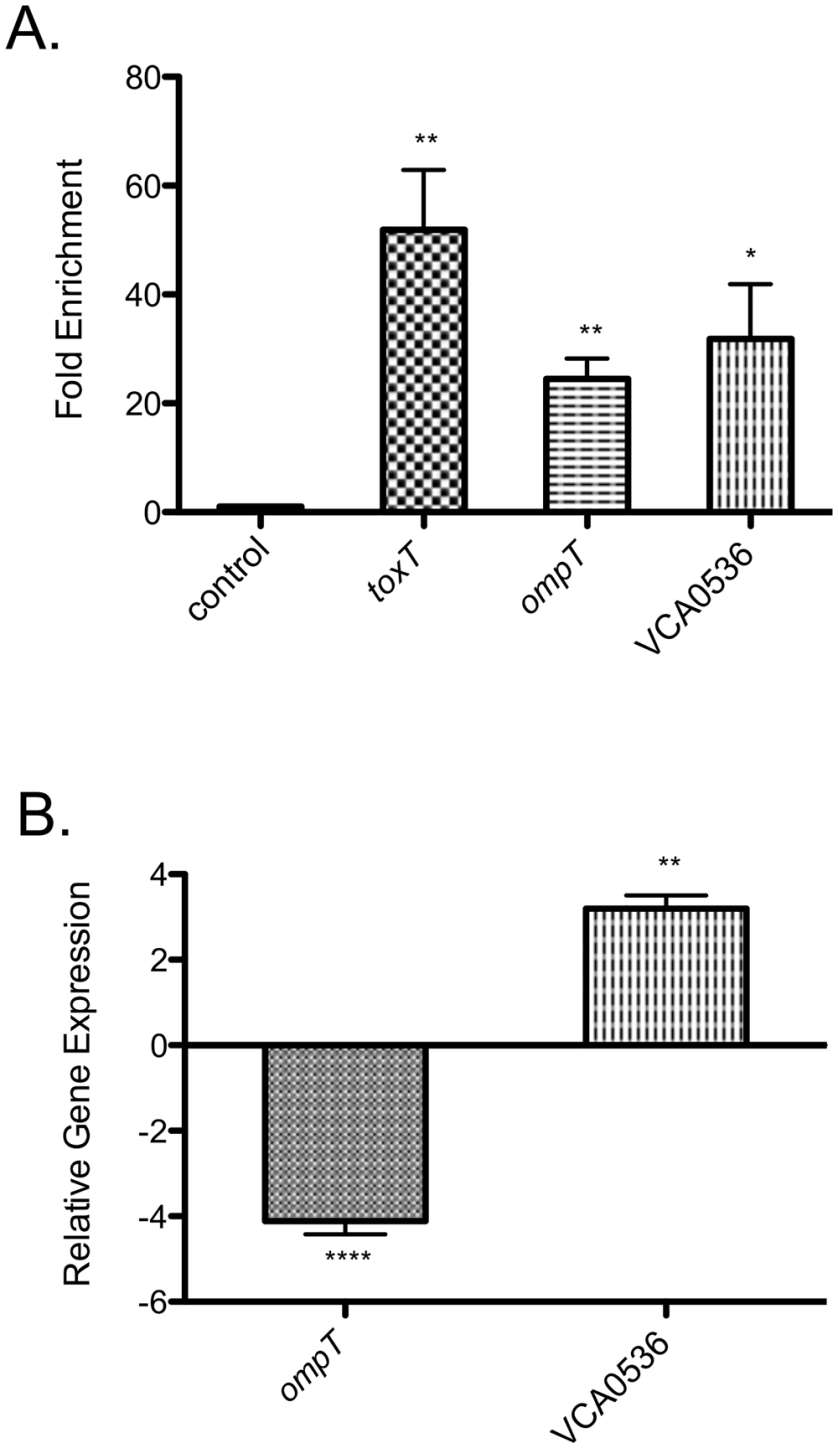
TcpP directly regulates *toxT*, *ompT*, and VCA0536. (A) TcpP ChIP enrichment of the promoter regions of *toxT*, *ompT*, and VCA0536. Enrichment of a non-TcpP-dependent promoter *icd* is shown as a control. TcpP enrichment of the promoter regions of *toxT*, *ompT*, and VCA0536 is statistically significant compared to the control. **p < 0.01; *p < 0.05, unpaired two-tailed Student’s *t* test. (B) qRT-PCR analysis of *ompT* and VCA0536 gene expression. The expression level of these genes in the Δ*tcpPH*+p*tcpPH* strain is shown, normalized to expression levels in the Δ*tcpPH*+vector control strain, which was set at 1. *ompT* expression is decreased in Δ*tcpPH*+p*tcpPH* compared to the control strain, while VCA0536 expression is increased in Δ*tcpPH*+p*tcpPH* compared to the control strain. ****p < 0.0001; **p < 0.005, unpaired two-tailed Student’s *t* test. Mean with standard error of the mean (SEM) is shown.

To define the regulon directly controlled by TcpP, we performed ChIP-seq in a similar manner as for ToxR. *tcpP* expression levels are shown in S1B Fig. qPCR analysis of TcpP ChIP DNA showed that the V5-tagged TcpP bound the *toxT* promoter, but not to a negative control locus (Fig 5A). In stark contrast to ToxR (and despite its relatively weak predicted binding motif constraints), our ChIP-seq analysis identified only three TcpP peaks in the entire *V*. *cholerae* genome (Table 2). We identified a strong TcpP peak upstream of *toxT*, agreeing with our initial validation of our TcpP construct (Fig 5A). A schematic of ChIP-seq DNA enrichment at this site is shown in S3 Fig. In addition, we identified TcpP peaks upstream of VC1854 (*ompT*) and hypothetical gene VCA0536. qPCR of TcpP ChIP DNA validated our sequencing data, showing TcpP binding of *ompT* and VCA0536 promoter regions, but not a negative control locus (Fig 5A). Enrichment of TcpP at *ompT* and VCA0536 promoter regions was similar to enrichment at the *toxT* promoter.

**Table 2:** Genes associated with TcpP ChIP-seq binding sites.

Gene	Function	TcpP Regulated^a^
VC0838 (*toxT*)	Virulence transcriptional regulator	Yes (33)
VC1854 (*ompT*)	Outer membrane protein	Yes (33)
VCA0536	Hypothetical protein	-

^a^citation for previously reported TcpP regulation is shown in brackets

Microarray analysis previously suggested TcpP can repress *ompT* expression [33]. Supporting this observation, we found that ectopic expression of *tcpPH* in a Δ*tcpPH* mutant repressed *ompT* expression compared with the empty plasmid control (Fig 5B). Along with *toxT*, *ompT* is now the second gene recognized as co-regulated by ToxR and TcpP. Moreover, TcpP repression of *ompT* shows that like ToxR, TcpP can act as either a transcriptional activator or repressor. VCA0536 has not previously been associated with TcpP regulation. VCA0536 encodes a putative cyclic di-GMP phospodiesterase that was found to be expressed *in vivo* by IVIAT [78], and is affected by the biofilm regulator VpsT [57]. Induction of *tcpPH* activated VCA0536 expression compared to the empty plasmid control (Fig 5B), supporting direct positive regulation by TcpP. Our results show that TcpP does regulate genes in addition to *toxT*, but does not share global regulation with ToxR despite similar predicted binding requirements.

### ToxR and TcpP binding motif analysis

We computationally scanned seven *V*. *cholerae* genomes, including both El Tor and Classical strains, for previously determined ToxR (TNAAA-N_5_-TNAAA) and TcpP (TGTAA-N_6_-TGTAA) binding motifs [15,20,77] using FIMO motif search software [79]. We used a cut-off p-value of < 0.0001 to identify significant sequence matches. For each motif, we identified many more matching sites in the genomes than were identified in their respective ChIP-seq analysis (S3 Table). This suggests that while primary DNA structure is undoubtedly important for ToxR and TcpP binding, the motif sequences alone are not sufficient to explain the selectivity of ToxR and TcpP binding *in vivo*

These motifs were constructed based on a small set of binding locations; four for ToxR and only 1 for TcpP. To attempt to improve the specificity of these motifs, we analyzed our ChIP-seq data sets for ToxR and TcpP binding site motif sequences using GLAM2 motif predication software [80,81]. We screened motifs generated through our analysis by determining if they overlapped with experimentally proven binding sites for TcpP in the *toxT* promoter, and for ToxR in the *toxT*, *ompU*, and *ompT* promoters. For ToxR and TcpP, we analyzed their respective ChIP-seq data sets as a whole and as peaks found on genomic islands compared to peaks found on the progenitor genome.

**Fig 6:**
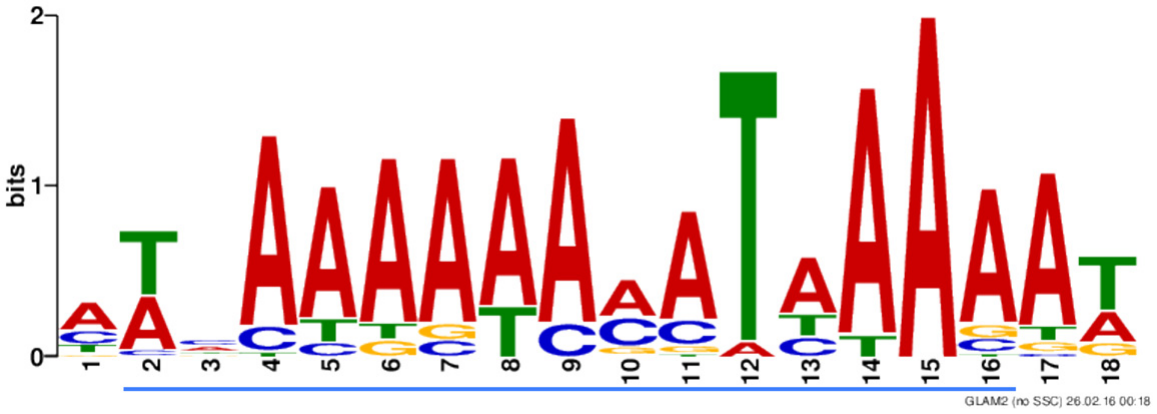
Predicted ToxR binding motif from ChIP-seq data. Sequences within ToxR ChIP-Seq peaks were used as input to identify a potential ToxR binding motif using GLAM2 software. This motif predicted from our ChIP-Seq data contains the previously established ToxR consensus sequence (TNAAA-N_5_-TNAAA) within it, which corresponds to the sequence underlined in blue. Larger letters indicate stronger base preferences at those sites.

The *V*. *cholerae* N16961 genome has an average GC-content of 47% [50]. ToxR ChIP peak sequences found in genomic islands and on the progenitor genome had lower average GC-contents of 38% and 42% respectively. Using all ToxR ChIP peak sequences, we were able to generate a motif that overlapped the previously published sequence important for ToxR binding and regulation of the *toxT*, *ompU*, and *ompT* promoters (Fig 6). This motif resembles the previously published motif and, like it, showed low sequence complexity and low GC-content. We computationally scanned seven *V*. *cholerae* genomes for this new motif using FIMO and again found it present more times throughout the genome than were identified by our ToxR ChIP-seq analysis (S3 Table). Use of this new motif alone also appears insufficient to predict locations bound by ToxR *in vivo*. We were unable to identify a TcpP binding motif from our ChIP peak dataset that also overlapped TcpP’s known binding site in the *toxT* promoter.

## Discussion

**Fig 7:**
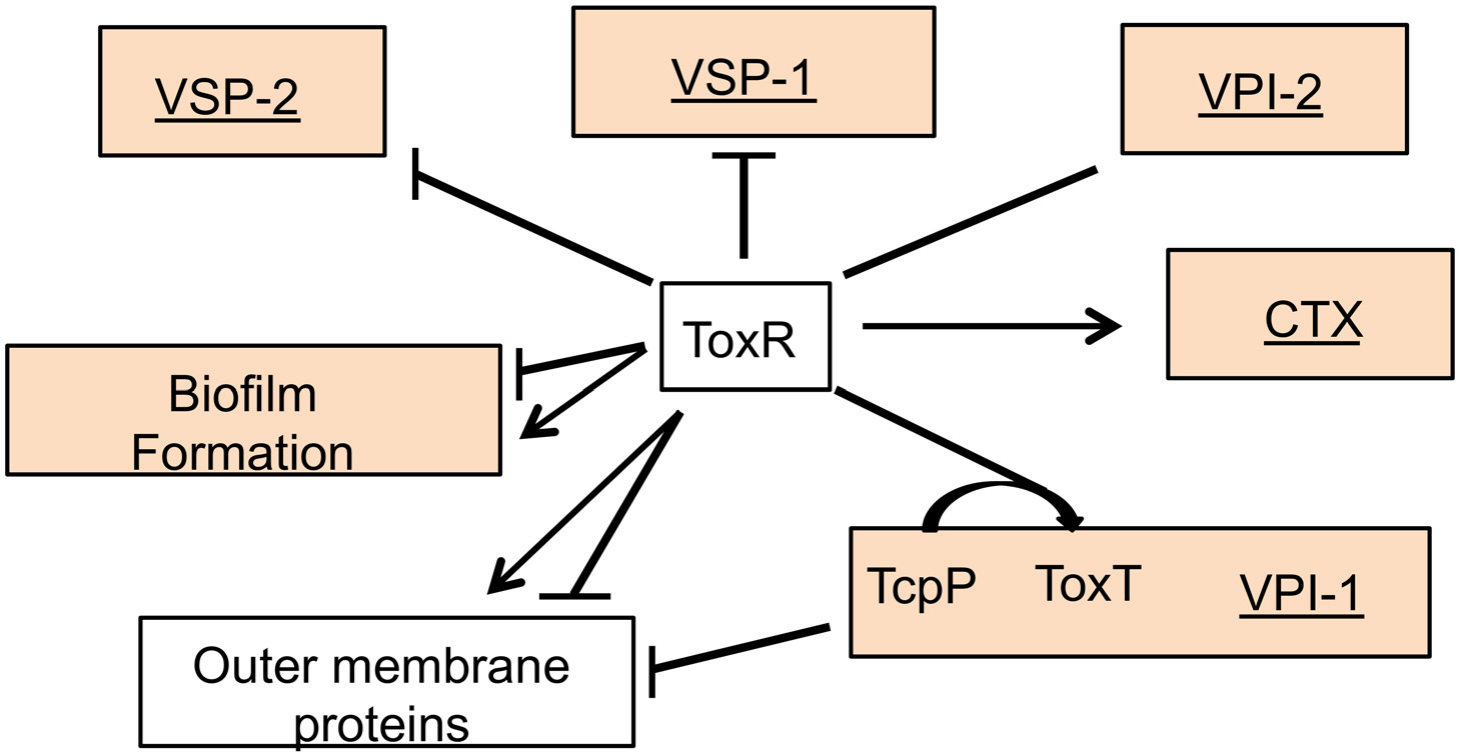
The direct ToxR regulon and its relationship to H-NS regulation. Genes clustered by general function or by location on acquired elements under ToxR and H-NS regulation are shown. Horizontally acquired elements are underlined. Arrows indicate positive regulation and perpendicular lines indicate negative regulation. Clusters containing genes bound by H-NS are shaded.

Our results indicate that ToxR directly controls a much larger gene set than previously recognized. This expands our understanding of virulence control and biofilm formation, and implicates ToxR as a broad regulator of acquired genetic information (Fig 7).

ToxR expression level and activity are regulated by many environmental signals [26–28,82,83]. ToxR also competes and interacts with other proteins to control transcription of target genes [29–31]. These factors likely allow *V*. *cholerae* to differentially control subsets of the ToxR regulon depending on the environmental conditions. The exact protein levels and activity of ToxR during each stage of infection or in biofilm development are unclear. In an attempt to overcome unknown environmental signals and broadly identify genomic sites for ToxR binding, we chose to use ectopic ToxR expression. This approach allows reproducible induction and immunoprecipitation of ToxR without prior knowledge of all the factors that may control its expression, and has proven effective for elucidating transcription factor regulons in *V*. *cholerae* and other bacteria [37–39].

A concern of this approach is that ectopic expression of ToxR or TcpP may cause aberrant binding or transcriptional regulation. While this remains a possibility, theoretical [84] and experimental studies [37,38,40] indicate that transcription factor overexpression does not lead to significant off target binding *in vivo*. Supporting our approach, the 35 ChIP loci we identified for ToxR is relatively small compared to many other prokaryotic ChIP-seq studies, which identified anywhere from several dozen to several hundred binding sites for other transcription factors [40,85–87]. Also, the *ctxA* promoter has been shown to bind ToxR *in vitro*, but the *in vivo* relevance of this is uncertain [18–20]. We did not identify this interaction with ChIP-seq, supporting that the expression level of ToxR used in our study did not promote ToxR binding to all available sites *in vivo*.

Our results indicate that ToxR regulation extends to all four *V*. *cholerae* pathogenicity islands, including VSP-1 and VSP-2, which genetically define seventh pandemic strains. The ability of ToxR to regulate new VSP-1 and VSP-2 functions along with existing cellular processes may have helped promote the emergence of 7^th^ pandemic strains. We identified a potential role for ToxR-regulated VSP-1 gene VC0176 in host colonization. VC0176 expression was found to be upregulated during intestinal colonization of the infant mouse model [88]. However, ToxR represses VC0176 expression and deletion of VC0176 results in a colonization defect. This suggests that ToxR may act on VC0176 to limit *V*. *cholerae* colonization at some point during the infection cycle, possibly in preparation for exiting the host. This is similar to the recent observation that ToxR can downregulate virulence gene expression through its regulation of *leuO* [51]. The ability of ToxR to gain direct control over VSP-1 and integrate it with existing virulence networks may have potentiated exploitation of VSP-1 gene functions and promoted the emergence of 7^th^ pandemic strains.

Our analysis identified additional ToxR regulated genes encoded on the progenitor genome, including those that function in biofilm formation. The positive regulation of *vpsL* by ToxR most adequately explains the defect in biofilm formation of the Δ*toxRS* mutant under our conditions. *vpsL* is the first gene in the vps-II operon [44,55,58]. Thus, ToxR activity likely influences additional genes downstream of *vpsL* that are also important for biofilm formation. This model would also help explain how the deletion of *hns* elevates the Δ*toxRS* biofilm defect. The regulatory relationship between *toxR*, *ryhB*, and VC1599 is less straightforward, but may be relevant for biofilm formation under different environmental conditions. ToxR regulation of *ryhB* and VC1599 could also be important for other aspects of *V*. *cholerae* biology, such as iron regulation, in which *ryhB* figures prominently. Deletion of *toxR* was recently shown to enhance biofilm formation of *V*. *cholerae* strain A1552 through an unknown mechanism in a standing culture in a silica tube [89]. The differences between those results and ours may be due to differences in assay conditions or, more likely, strain differences. Our studies used strain C6706, while Valeru *et al*. used strain A1552. Phenotypic differences between these strains have previously been observed with competency and *Vibrio* polysaccharide regulation, and may be attributed to strain variation in cAMP-CRP or quorum-sensing regulation [90,91]. It is worth noting that biofilms can enhance gene transfer [92–97] and ToxR is involved in both biofilm formation and broad regulation of acquired genes. It will be interesting to test if ToxR also enhances gene transfer or stability of acquired elements.

Our results provide genetic evidence that the master regulator ToxR antagonizes H-NS activity at sites across the genome to affect important phenotypes. This result is consistent with previous studies describing interactions between H-NS, ToxT, and ToxR in regulating expression of *toxT*, *tcpA* and *ctx* [31,67]. Importantly, we demonstrate that deleting *hns* eliminates the requirement of ToxR for host colonization in modern 7^th^ pandemic *V*. *cholerae*. This result suggests that the major role of ToxR in virulence is to antagonize H-NS repression of colonization factors.

The mechanism of ToxR antagonism is unclear. Rather than one mechanism, the way in which ToxR and H-NS interact may vary with genomic location. Moreover, since H-NS gene silencing is regulated by environmental factors [69,72], the interaction between H-NS and ToxR may change as *V*. *cholerae* cycles between host and environmental reservoirs. Our ChIP-seq analysis shows that ToxR and H-NS share certain binding locations across genome (such as the *toxT* promoter), and induction of *toxRS* results in decreased DNA binding of H-NS. ToxR may directly compete with and displace H-NS at shared binding sites, as has been suggested for other H-NS/transcription factor interactions [67,98]. Rather than sequence alone, H-NS has an affinity for DNA structure, favoring the binding of curved DNA [99–101], and is known to form nucleoprotein filaments that promote DNA silencing [102]. ToxR may bind and alter DNA topology near H-NS, which could destabilize its interactions with DNA. Alternatively, ToxR may directly interact with H-NS and destabilize its DNA association, as has been shown for phage protein Arn [103].

Understanding how ToxR recognizes its target DNA sequences will be important in deciphering its antagonism of H-NS. Our analysis of ChIP-seq peaks identified an expanded ToxR consensus DNA motif that may facilitate its DNA binding. However, the large number of locations of this motif in the genome compared to the number of ToxR binding sites we identified suggests that our motif is still inadequate to predict ToxR binding specificity *in vivo* alone. It is possible that a primary structure of A, T, G, and C that does dictate ToxR binding was left undiscovered by our analysis. Differences between predicted and actual *in vivo* binding sites were also observed for ToxT, which also has a low GC-content and low-complexity consensus motif. Computationally, the ToxT consensus motif (toxbox) maps to a large number of locations across the *V*. *cholerae* genome [104]. However, *in vitro* biochemical interactions between purified ToxT and fragmented *V*. *cholerae* genome identified just 199 ToxT binding sites [105]. Subsequently, *in vivo* ChIP-seq identified and validated only seven of these ToxT binding sites, which is in line with transcriptome studies of ToxT regulated genes [33,38].

In eukaryotic gene regulation, factors in addition to linear DNA sequence, including topology, partner proteins, and DNA localization, all contribute to *in vivo* selectivity of transcription factor DNA binding [106,107]. Analysis of 119 transcription factors from the ENCODE project database has shown up to 99% of motif locations in a genome are not bound by their respective transcription factor [108]. Like H-NS, ToxR has a propensity to bind low GC-content DNA. Thus, ToxR binding may also use DNA topology in addition to sequence. ToxR is also unique in that it is a membrane-bound transcription factor. ToxR’s localization may limit its access to genome locations in a packed nucleoid. Super-resolution microscopy has suggested that H-NS sequesters bound DNA into two compact clusters per chromosome in *E*. *coli* [109]. Similar nucleoid structuring in *V*. *cholerae* could also act to limit ToxR access to all genomic locations. Future localization and chromosome conformation capture studies may yield important information on factors in addition to primary DNA structure that dictate how ToxR reaches its target sequences. Continued research to understand how ToxR finds its regulatory targets may provide insight into the evolutionary trajectory of *V*. *cholerae* and its potential for future acquisition of foreign genes.

## Methods

### Ethics statement

The animal experiments were performed with protocols approved by the University of Texas at Austin, Institutional Animal Care and Use Committee. Protocol number AUP-2013-00052. The University of Texas at Austin animal management program is accredited by the Association for the Assessment and Accreditation of Laboratory Animal Care, International (AAALAC), and meets National Institutes of Health standards as set forth in the Guide for the Care and Use of Laboratory Animals (DHHS Publication No. (NIH) 85–23 Revised 1996).

### Bacterial strains

Strains and plasmids are listed in S4 Table. Strains were grown in Luria Broth (LB; rich medium). The following antibiotic concentrations were used: carbenicillin 75 μg/mL, kanamycin 25 μg/mL, streptomycin 100 μg/mL and chloramphenicol 2.5 μg/mL for *V*. *cholerae* and 10 μg/mL for *E*. *coli*. Arabinose was used at 0.2% for induction. X-gal was used at 40 μg/mL.

### DNA manipulations

All cloning products were sequence-verified, and the nucleotide sequences of all primers used for cloning are listed in S5 Table. For in-frame gene deletions of *toxRS*, *tcpPH*, VC1599, VCA0536, *vpsL* and H-NS, genomic DNA surrounding the respective gene was amplified by crossover PCR and cloned into pWM91 or pSSK10 for subsequent *sacB* mediated allelic exchange as described [110,111]. For complementation constructs, the respective gene was amplified from chromosomal DNA and cloned into plasmid pBAD18 or pWKS30 [112,113]. For genes cloned into pWKS30, the respective native promoter was also included. Full length ToxR and TcpP were cloned into pBAD18 with C-terminal 3XV5 tags as previously described [37–39]. Genes cloned into pBAD18 were induced by adding arabinose to the growth medium.

### Biofilm assays

Biofilm assays were performed essentially as described [114]. *V*. *cholerae* C6706 wild-type and mutants strains where grown overnight on LB agar plates. Each strain was back-diluted in a 5 mL culture of LB and grown to mid-log phase. The culture was then diluted 1:100 in fresh LB, and 100μL of the diluted culture was added to a round-bottom PVC microtiter plate in replicates of three. Strains were allowed to grow for 22 hours at 30°C. Planktonic cells were removed, and bound cells were washed twice with 200 μL sterile water and then stained with 0.1% crystal violet for 15 mins. Stain was removed and cells were washed three times with 200 μL PBS and allowed to air dry for 15–30 mins. Stain was then solubilized with 200 μL 95% ethanol for 15 mins. Finally, 125 μL of solubilized stain was transferred to a new 96-well, flat bottom polystyrene plate. The optical density was measured at 595 nm using a SpectraMax Plus384 absorbance microplate reader with SOFTmax Pro v6.2.2 software.

### Chromatin immunoprecipitation

ChIP was performed as previously described [37,38]. 50 mL of exponentially growing culture in LB was induced with 0.1% arabinose for 30 min at 37°C. No induction was required for H-NS ChIP. Formaldehyde was added to 1% final concentration and incubated at 25°C for 20 min with occasional swirling. Crosslinking was quenched by adding glycine to 0.5 M. Cell pellets were washed in 1X TBS and resuspended in lysis buffer (10 mM Tris pH 8.0, 100 mM NaCl, 1 mM EDTA, 0.5 mM EGTA, 0.1% DOC, 0.5% N-lauroylsarcosine) + protease inhibitor cocktail (Sigma) and 1 mg/mL lysozyme and incubated at 37°C for 30 min. The cells were sonicated 1X 30sec with a needle sonicator, and unlysed debris was pelleted by centrifugation. The lysate was sonicated for 20 min with a 10 s on/ 10 s off cycle (QSonica; www.sonicator.com). Sheared samples had an average DNA fragment size of ~300bp with a spread of 50-800bp. A sample was taken as a non-immunoprecipitated input control for sequencing. Following clarification by centrifugation, 1/10 volume of 10% Triton X-100 in lysis buffer was added to each sample followed by 100 μl of Dynal-Protein G beads coated with anti-V5 monoclonal antibody (Sigma) and incubated overnight with rotation. The beads were washed 5X with ChIP RIPA buffer [50 mM HEPES pH 7.5, 500 mM LiCl, 1 mM EDTA, 1% NP40, 0.7% DOC], then 1X in TE + 50 mM NaCl and resuspended in 100 μL elution buffer [50 mM Tris-HCl, pH 7.5, 10 mM EDTA, 1% SDS]. Samples were incubated at 65°C for 30 min and the beads pelleted by centrifugation. Supernatants were incubated at 65°C overnight to reverse crosslinks. Samples were incubated with 8 μL of 10 mg/mL RNase A for 2 hr at 37°C, then 4 μL of 20 mg/ml proteinase K at 55°C for 2 hr, then purified. Experiments were repeated in at least biological duplicate. Sequencing sample preparation was performed as previously described [37].

### Sequence data processing

Samples were sequenced using Illumina HiSeq. Data processing for ChIP-seq was performed as previously described [37–39]. Sequence reads were aligned to the *V*. *cholerae* N16961 genome using CLC genomic workbench software. CLC genomic workbench ChIP-seq software was used to compare control input and experiment alignments to identify peak enrichment. Our DNA sonication method results in an average DNA fragment size of ~300bp with a spread of 50-800bp. A transcription factor can occupy the extreme ends of up to an 800bp fragment allowing a raw peak to be called that spans up to ~1600bp. We have reported these maximum raw coordinates in S1 Table (ToxR) and S2 Table (H-NS), without computational refinement. Peaks that were identified in both replicates were scored as real peaks.

### DNA binding motif analysis

All motif studies were performed using the MEME Suite of motif-based sequence analysis tools [79–81]. Genome scanning for motifs was performed with FIMO version 82 with a stringent p-value cut-off of <0.0001. FIMO returns sequences that match the input motif with a probability specified by the p-value. Identification of ToxR and TcpP binding motifs from ChIP-seq data was performed with both MEME and GLAM2. We analyzed the respective ChIP-seq data as a whole, and separated into peaks found on genomic islands compared to peaks found on the progenitor genome. We screened motifs generated through our analysis to determining if they overlapped with the biochemically proven binding sites for TcpP in the *toxT* promoter, and for ToxR in the *toxT*, *ompU*, and *ompT* promoters. We focused on identification of ungapped motifs. We did not identify a TcpP motif that meets our criteria. We identified a ToxR motif using GLAM2 present in all ToxR ChIP-seq peak sequences that met our criteria.

### Quantitative PCR

For ChIP-seq peak validation, relative abundance quantitative PCR (qPCR) was performed with Kapa Biosystems Sybr Fast One-Step qRT-PCR kit using 16S rDNA as the internal reference. Relative target levels were calculated using the ΔΔCt method, with normalization of ChIP targets to 16S rDNA signal [37]. For gene expression analysis, relative expression reverse-transcription quantitative PCR was performed with Applied Systems RNA-Ct one-step system. Relative expression levels were calculated using the ΔΔCt method, with normalization of gene targets to16S rRNA signals [37].

### Northern blots

RNA was prepared from logarithmic cultures in triplicate under the same growth conditions used for ChIP-seq. Equal amounts of total RNA were separated on a 6% TBE-urea gel and transferred to Hybond N membrane. After crosslinking and prehybridization, membranes were incubated with 100 pmol of 32P labeled probe. Washed membranes were exposed to film overnight. Bands were quantified by densitometry. RyhB and 5S probes are listed in S5 Table.

### Infant mouse colonization assays

A modified version of the protocol of Baselski and Parker [115] was performed for infection and recovery of all strains. Strains were grown on selective medium overnight at 37°C. Wild-type and mutant strains were mixed together in LB. 50 μL of this competition mixture (∼50,000 bacteria) was inoculated into a 5-day-old CD1 mouse pup (Charles River Company). One strain carried an active *lacZ* allele. Serial dilutions of the competition mixture were plated on selective medium and enumerated to determine the input ratio of wild type and mutant strain. After incubation at 30°C for 18 hr the mouse pups were sacrificed and small intestines were removed and homogenized in 10 mL of LB. Serial dilutions were plated in LB + Sm100 + Xgal and enumerated to determine the output ratio of wild-type and mutant strain. The competitive index for each mutant is defined as the output ratio of mutant/wild-type strain divided by the input ratio of mutant/wild-type strain. Statistical significance was determined by comparing the resulting ratio to the ratio of WT versus WT *lacZ*−. At least five mice were tested for each mutant.

### Statistical analysis

Data were analyzed using GraphPad Prism 5 Software. Statistical significance between two groups was assessed using an unpaired two-tailed Student’s *t* test. Statistical significance when comparing more than two groups was assessed using a One-Way ANOVA analysis followed by a Tukey’s multiple comparison post-test. Standard error of the mean (SEM) is shown.

### Data deposition

The sequence data have been deposited with the NCBI’s Gene Expression Omnibus under Accession Number GSE72474.

## Supporting Information

S1 Fig ToxR and TcpP expression levels used for ChIP-seq. A) Western blot for ToxR-3XV5 following arabinose induction. An anti-ToxR antibody shows expression of endogenous and plasmid borne ToxR levels in wild type carrying empty vector (pBAD18Cm) and wild type carrying p*toxR*-3XV5 on pBAD18Cm following arabinose induction. The 3XV5 tag adds 4.5kD in molecular mass to ToxR. An anti-V5 antibody shows expression levels of ToxR-V5 alone. Arabinose induction of increases ToxR expression levels 5.3 ± 0.01 fold relative to wild type (mean with standard error of the mean (SEM) reported). p < 0.001, unpaired two-tailed Student’s *t* test. RpoB is shown as a loading control. All samples were processed on the same gel with biological triplicate samples. B) *tcpP* mRNA levels following arabinose induction of TcpP-3XV5. *tcpP* expression was significantly greater in the WT+*tcpP*–3XV5 strain relative to the WT+pBAD18cm control strain. ***p < 0.001, unpaired two-tailed Student’s *t* test. Mean with standard error of the mean (SEM) is shown.
(TIF)

S2 Fig ToxR ChIP enrichment at the promoter regions of *toxT*, *ompT* and *ompU*. ToxR ChIP fold enrichment of the promoter regions of *toxT*, *ompT*, and *ompU* was determined by qPCR relative to the enrichment of a non-ToxR-dependent *icd* promoter, shown as a negative control. ToxR enrichment of the promoter regions of *toxT*, *ompT* and *ompU* is statistically significant compared to the control. ****p < 0.0001; ***p < 0.001, unpaired two-tailed Student’s *t* test. Mean with standard error of the mean (SEM) is shown.
(TIF)

S3 Fig Heat maps of raw ToxR and TcpP ChIP-seq read alignments to the N16961 *V*. *cholerae* genome. The heat map shows enrichment (red) of DNA reads at selected ToxR and TcpP binding locations. Schematic of raw ToxR ChIP-seq read alignment proximal to (A) *ompU* (VC0633), (B) *ryhB* (between VC0106-VC0107), and (C) *toxT* (VC0838). (D) Schematic of raw TcpP ChIP-seq read alignment proximal to *toxT* (VC0838).
(TIF)

S4 Fig Gene expression analysis comparing wild type to Δ*toxRS* mutant at select loci. qRT-PCR analysis of *vpsL*, VC1599, VC0176, VC0178, and VC0493 gene expression. The expression level of these genes in the Δ*toxRS* strain is shown, normalized to expression levels in the wild type strain, which was set at 1. The expression levels of these genes in the Δ*toxRS* mutant strain are not significantly different relative to expression levels in the wild type strain under this condition by unpaired two-tailed Student’s *t* test.
(TIF)

S5 Fig Complementation of biofilm phenotypes in rich medium. Biofilm assays were performed as described in the methods. All biofilm measurements were normalized to the wild-type strain carrying the control plasmid pWKS30, which was set at 1. Each mutant strain carrying the empty vector show a difference in biofilm production compared to the WT+pWKS30 strain; p < 0.05 determined by One-Way ANOVA analysis followed by a Tukey’s multiple comparison post-test. *ΔtoxRS+*pWKS30, ΔVC1599*+*pWKS30, Δ*ryhB+*pWKS30, and Δ*vpsL+*pWKS30 have a defect in biofilm formation compared to the respective complemented mutant strains Δ*toxRS+*p*toxRS*, ΔVC1599*+*p1599, Δ*ryhB+*p*ryhB*, and Δ*vpsL+*p*vpsL*. Statistical significance was determined by One-Way ANOVA analysis followed by a Tukey’s multiple comparison post-test, ***p < 0.001; **p < 0.01. Mean with standard error of the mean (SEM) is shown.
(TIF)

S6 Fig Quantification of biofilm formation by double mutants in rich medium. Biofilm assays were performed as described in the methods. All biofilm measurements were normalized to the Δ*toxRS* mutant, which was set to 1. Δ*toxRS*ΔVC1599, Δ*toxRS*Δ*ryhB*::*kan* ^*R*^ and Δ*toxRS*Δ*vpsL* double mutant biofilm formation was not statistically significant compared to the Δ*toxRS* mutant, unpaired two-tailed Student’s *t* test.
(TIF)

S7 Fig Complementation of the VC0176 mouse colonization defect. Results of infant mouse colonization assays of each indicated strain competed against the wild type strain carrying empty vector. pWKS30 is the empty vector. p176 is the complementing plasmid expressing VC1076 from its native promoter. ΔVC0176+pWKS30 showed a defect in infant mouse colonization compared to the complemented strain ΔVC0176+p176. ****p < 0.0001, unpaired two-tailed Student’s *t* test.
(TIF)

S8 Fig Complementation of *toxRS* and *hns* mouse colonization phenotypes. Results of infant mouse colonization assays of each indicated strain competed against the wild type strain carrying empty vector. pWKS30 is the empty vector. p*toxRS* and p*hns* are complementing plasmids expressing *toxRS* and *hns* respectively under their native promoters. Δ*toxRS*+pWKS30 showed a defect in infant mouse colonization compared to the complemented strain Δ*toxRS+*p*toxRS*, as well as Δ*toxRS*Δ*hns+*pWKS30. Δ*toxRS*Δ*hns+*p*hns* showed a defect in colonization of the infant mouse intestine compared to Δ*toxRS*Δ*hns+*pWKS30, as well as Δ*toxRS+*p*toxRS*. Statistical significance was determined by One-Way ANOVA analysis followed by a Tukey’s multiple comparison post-test, ***p < 0.001.
(TIF)

S9 Fig Complementation of *hns* biofilm phenotypes in rich medium. Biofilm assays were performed as described in the methods. All biofilm measurements were normalized to the wild type strain carrying the empty vector pWKS30, which was set to 1. p*hns* is the plasmid encoding *hns* expressed from its native promoter. Each mutant showed increased biofilm production compared to the WT+pWKS30 strain. Δ*hns+*p*hns* showed a defect in biofilm formation compared to *Δhns+*pWKS30. Δ*toxRS*Δ*hns+*p*hns* showed a defect in biofilm formation compared to Δ*toxRS*Δ*hns+*pWKS30. Statistical significance was determined by One-Way ANOVA analysis followed by a Tukey’s multiple comparison post-test, ***p < 0.001. Standard error of the mean (SEM) is shown.
(TIF)

S1 Table Coordinates of raw ToxR ChIP-seq peaks and associated genes. (DOCX)

S2 Table Coordinates of raw H-NS ChIP-seq peaks and associated genes. (XLSX)

S3 Table Number of DNA motif occurrences in *V*. *cholerae* genomes determined by FIMO. (XLSX)

S4 Table Strain and plasmid list. (DOCX)

S5 Table Primer list. (DOCX)
