# Supplementary figures and images for: ToxR Antagonizes H-NS Regulation of Horizontally Acquired Genes to Drive Host Colonization
Source: PLoS Pathog. 2016 Apr 12;12(4):e1005570. doi: 10.1371/journal.ppat.1005570 (PMC4829181; doi:10.1371/journal.ppat.1005570)

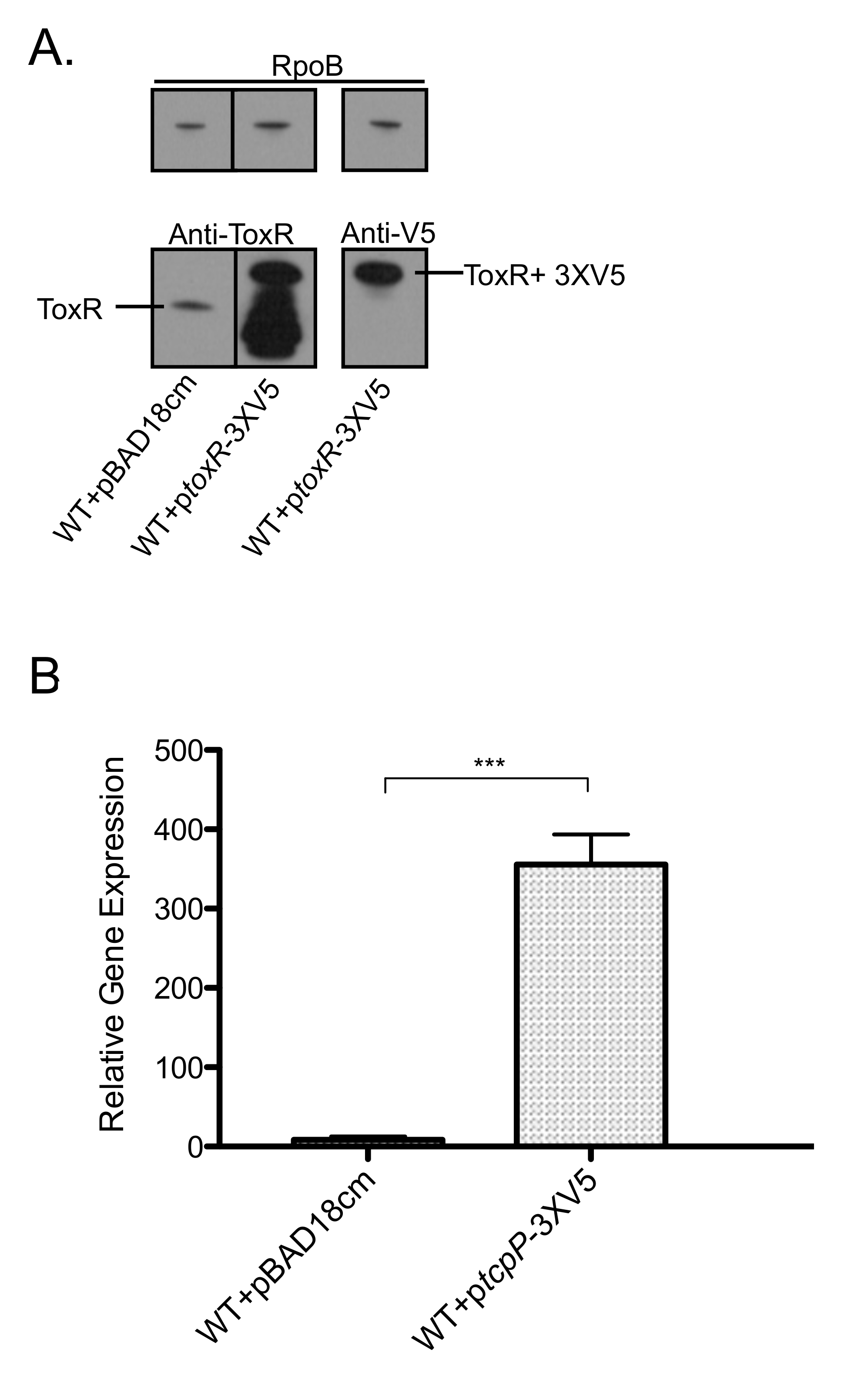

Supplement: S1 Fig — A) Western blot for ToxR-3XV5 following arabinose induction. An anti-ToxR antibody shows expression of endogenous and plasmid borne ToxR levels in wild type carrying empty vector (pBAD18Cm) and wild type carrying ptoxR-3XV5 on pBAD18Cm following arabinose induction. The 3XV5 tag adds 4.5kD in molecular mass to ToxR. An anti-V5 antibody shows expression levels of ToxR-V5 alone. Arabinose induction of increases ToxR expression levels 5.3 ± 0.01 fold relative to wild type (mean with standard error of the mean (SEM) reported). p < 0.001, unpaired two-tailed Student’s t test. RpoB is shown as a loading control. All samples were processed on the same gel with biological triplicate samples. B) tcpP mRNA levels following arabinose induction of TcpP-3XV5. tcpP expression was significantly greater in the WT+tcpP–3XV5 strain relative to the WT+pBAD18cm control strain. ***p < 0.001, unpaired two-tailed Student’s t test. Mean with standard error of the mean (SEM) is shown. (TIF) [file ppat.1005570.s001.tif]

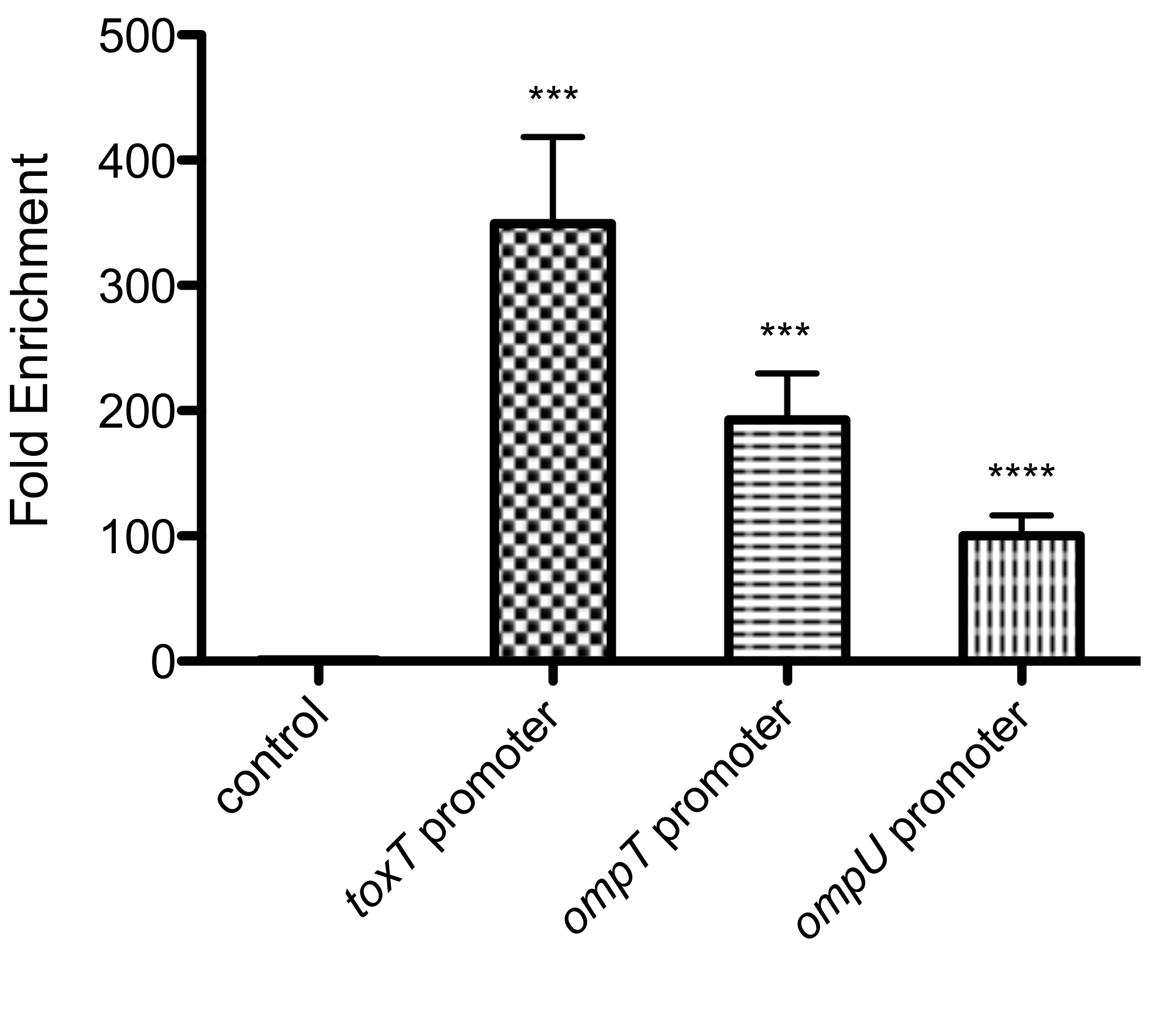

Supplement: S2 Fig — ToxR ChIP fold enrichment of the promoter regions of toxT, ompT, and ompU was determined by qPCR relative to the enrichment of a non-ToxR-dependent icd promoter, shown as a negative control. ToxR enrichment of the promoter regions of toxT, ompT and ompU is statistically significant compared to the control. ****p < 0.0001; ***p < 0.001, unpaired two-tailed Student’s t test. Mean with standard error of the mean (SEM) is shown. (TIF) [file ppat.1005570.s002.tif]

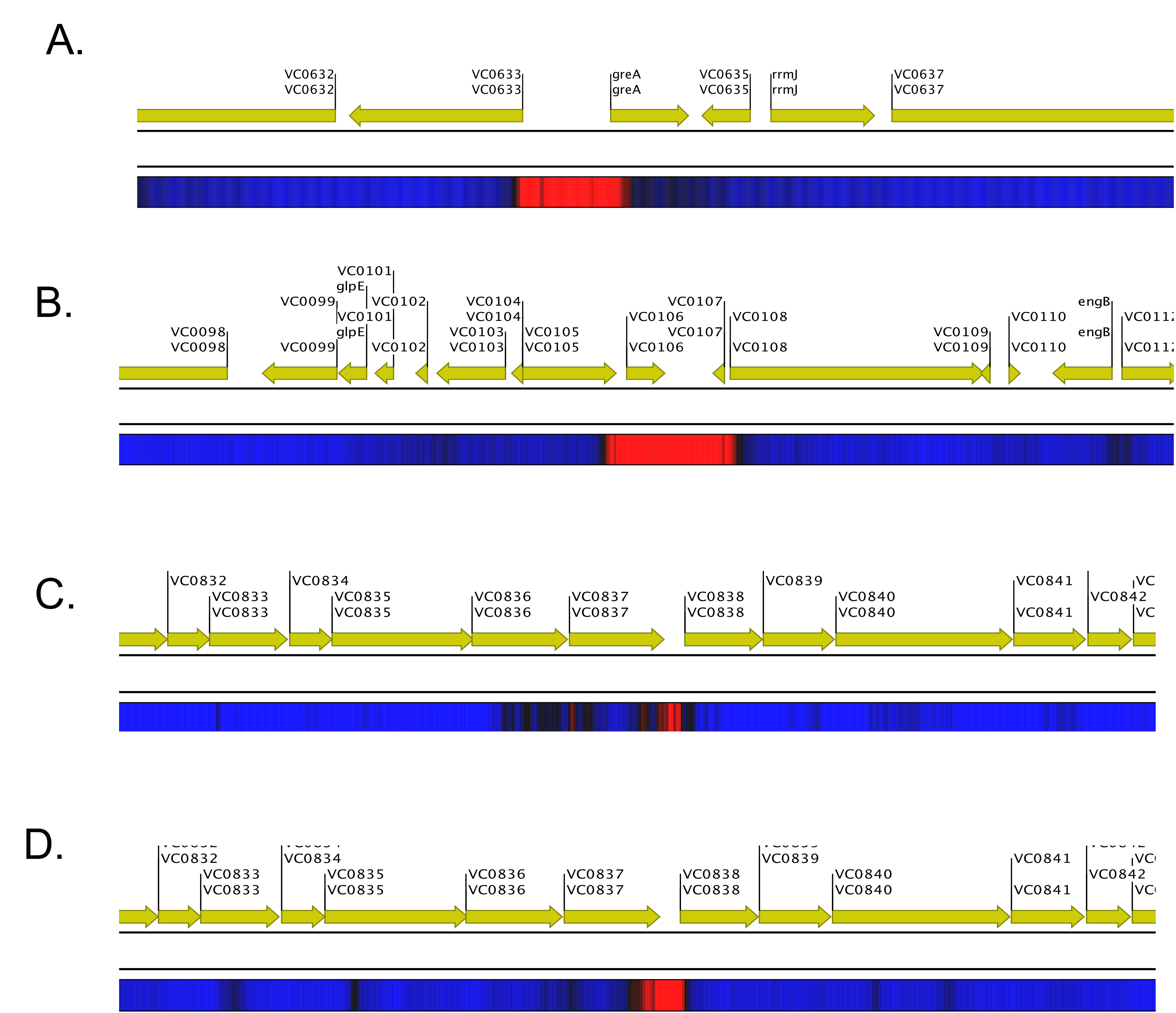

Supplement: S3 Fig — The heat map shows enrichment (red) of DNA reads at selected ToxR and TcpP binding locations. Schematic of raw ToxR ChIP-seq read alignment proximal to (A) ompU (VC0633), (B) ryhB (between VC0106-VC0107), and (C) toxT (VC0838). (D) Schematic of raw TcpP ChIP-seq read alignment proximal to toxT (VC0838). (TIF) [file ppat.1005570.s003.tif]

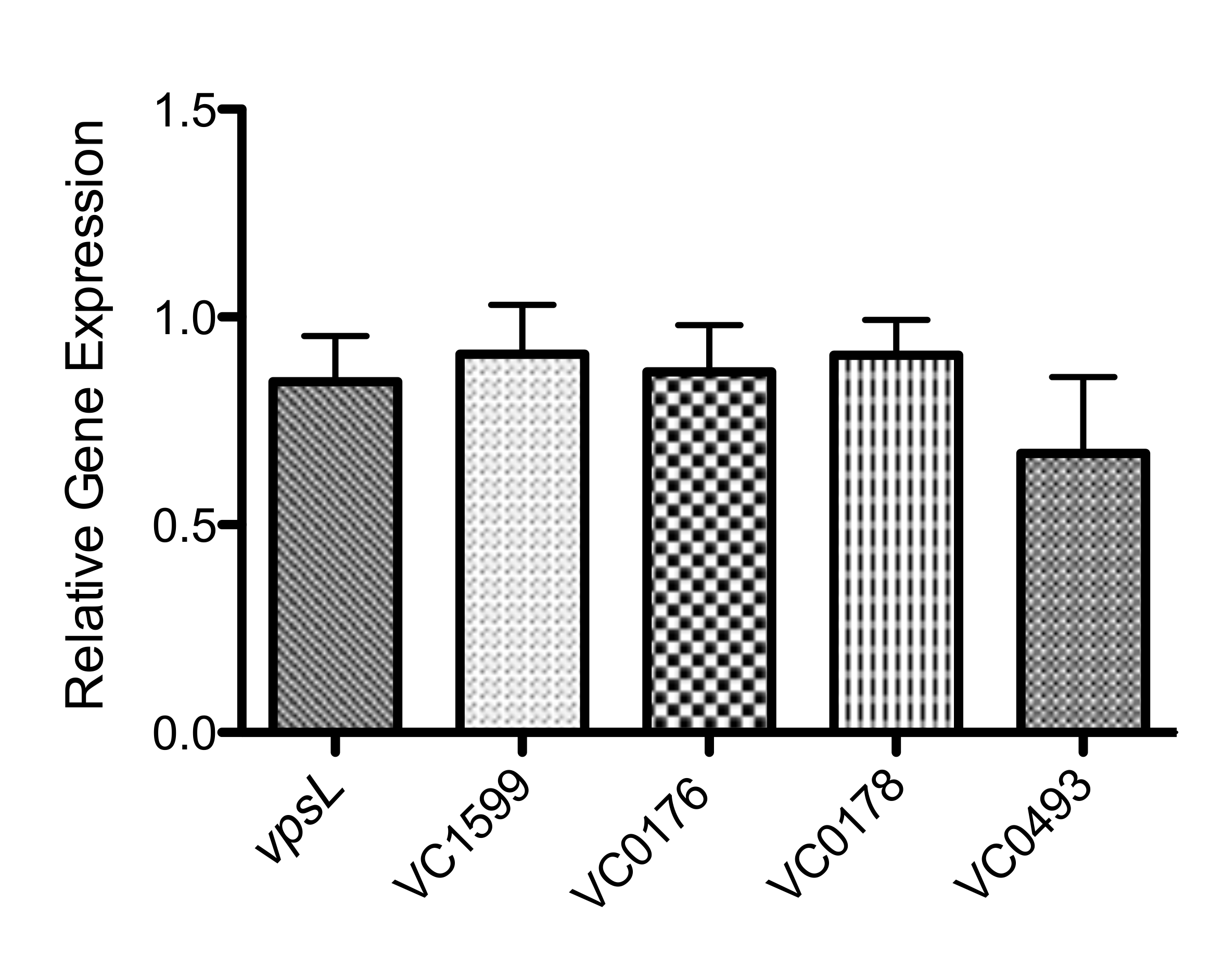

Supplement: S4 Fig — qRT-PCR analysis of vpsL, VC1599, VC0176, VC0178, and VC0493 gene expression. The expression level of these genes in the ΔtoxRS strain is shown, normalized to expression levels in the wild type strain, which was set at 1. The expression levels of these genes in the ΔtoxRS mutant strain are not significantly different relative to expression levels in the wild type strain under this condition by unpaired two-tailed Student’s t test. (TIF) [file ppat.1005570.s004.tif]

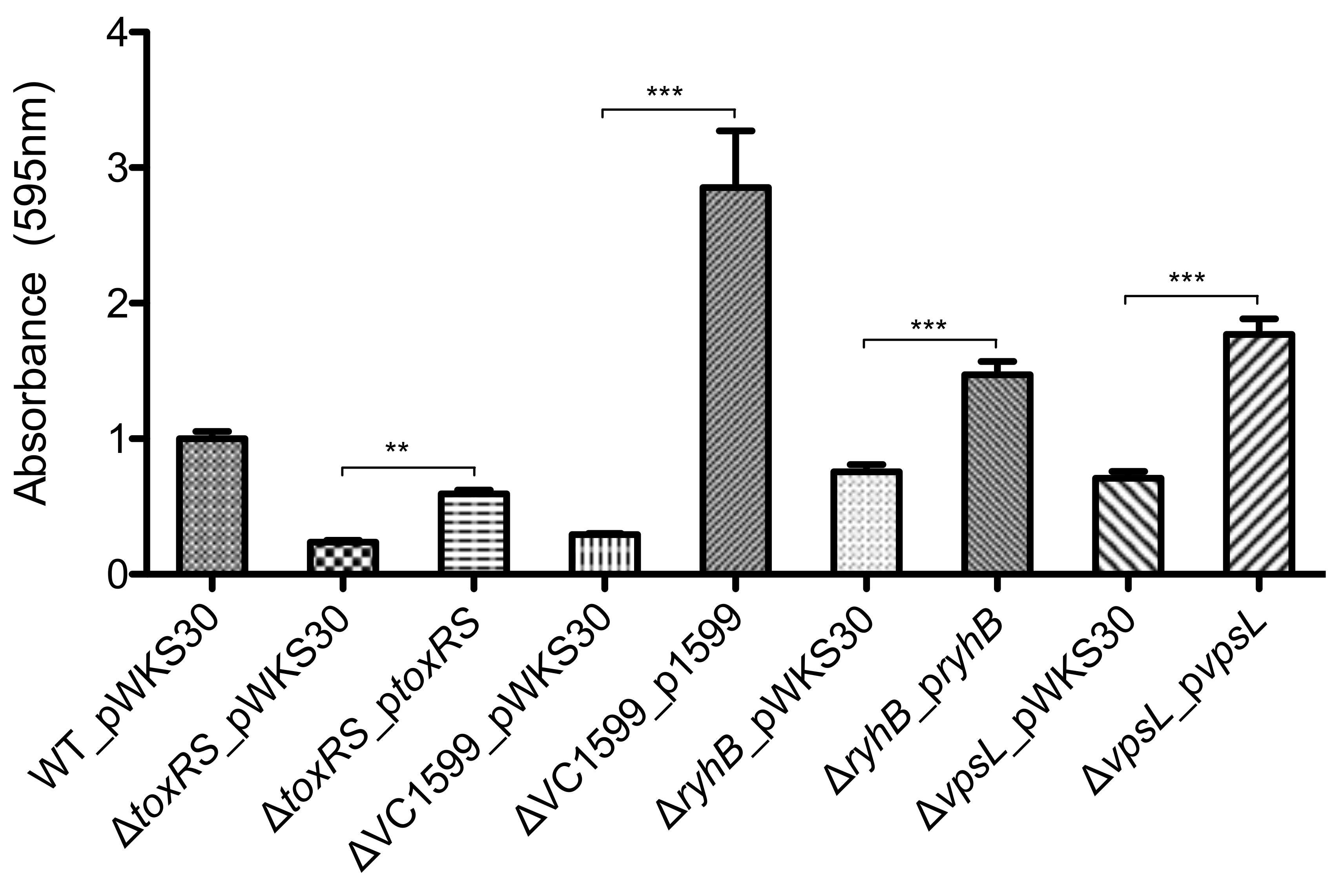

Supplement: S5 Fig — Biofilm assays were performed as described in the methods. All biofilm measurements were normalized to the wild-type strain carrying the control plasmid pWKS30, which was set at 1. Each mutant strain carrying the empty vector show a difference in biofilm production compared to the WT+pWKS30 strain; p < 0.05 determined by One-Way ANOVA analysis followed by a Tukey’s multiple comparison post-test. ΔtoxRS+pWKS30, ΔVC1599+pWKS30, ΔryhB+pWKS30, and ΔvpsL+pWKS30 have a defect in biofilm formation compared to the respective complemented mutant strains ΔtoxRS+ptoxRS, ΔVC1599+p1599, ΔryhB+pryhB, and ΔvpsL+pvpsL. Statistical significance was determined by One-Way ANOVA analysis followed by a Tukey’s multiple comparison post-test, ***p < 0.001; **p < 0.01. Mean with standard error of the mean (SEM) is shown. (TIF) [file ppat.1005570.s005.tif]

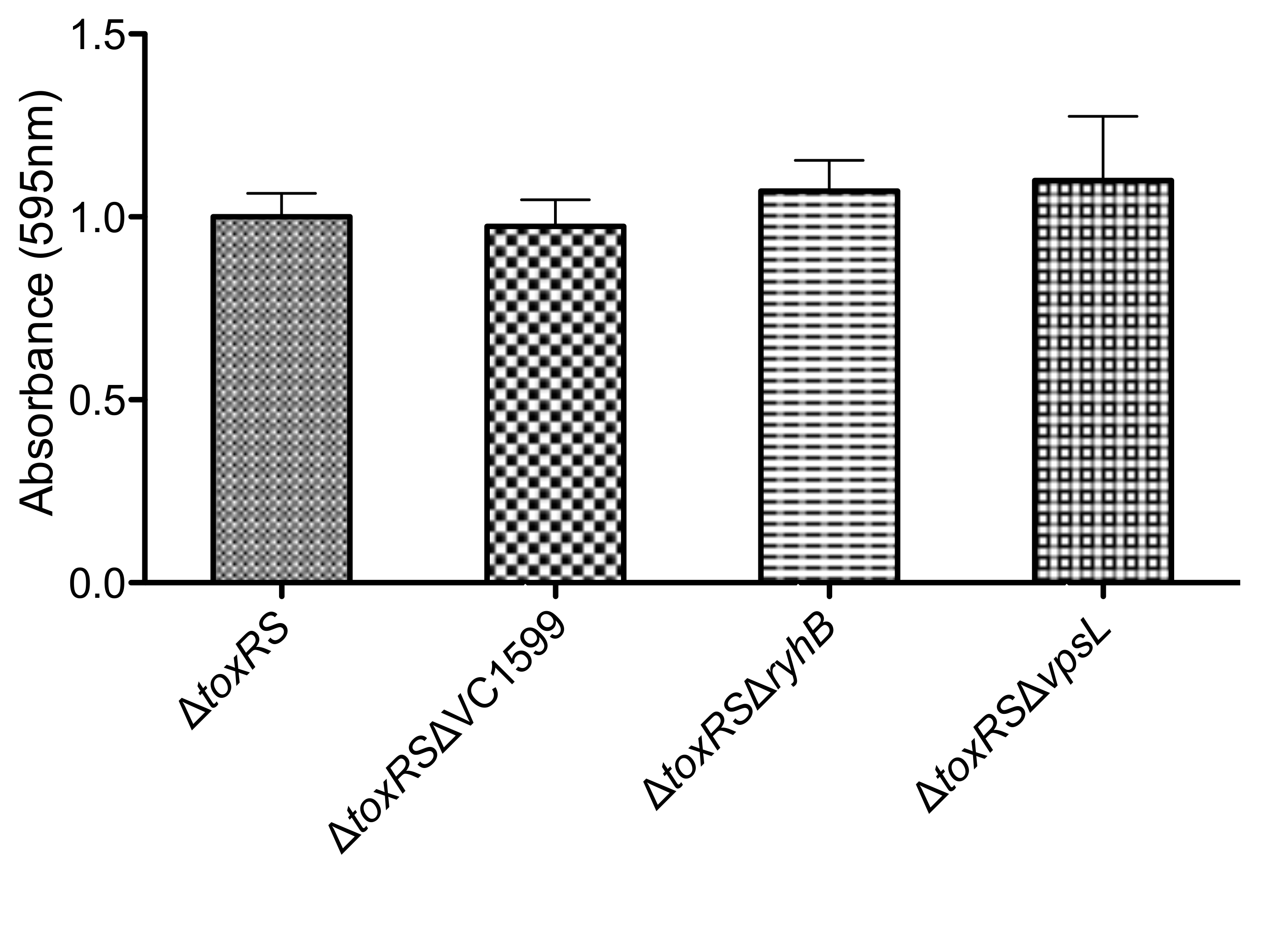

Supplement: S6 Fig — Biofilm assays were performed as described in the methods. All biofilm measurements were normalized to the ΔtoxRS mutant, which was set to 1. ΔtoxRSΔVC1599, ΔtoxRSΔryhB::kan R and ΔtoxRSΔvpsL double mutant biofilm formation was not statistically significant compared to the ΔtoxRS mutant, unpaired two-tailed Student’s t test. (TIF) [file ppat.1005570.s006.tif]

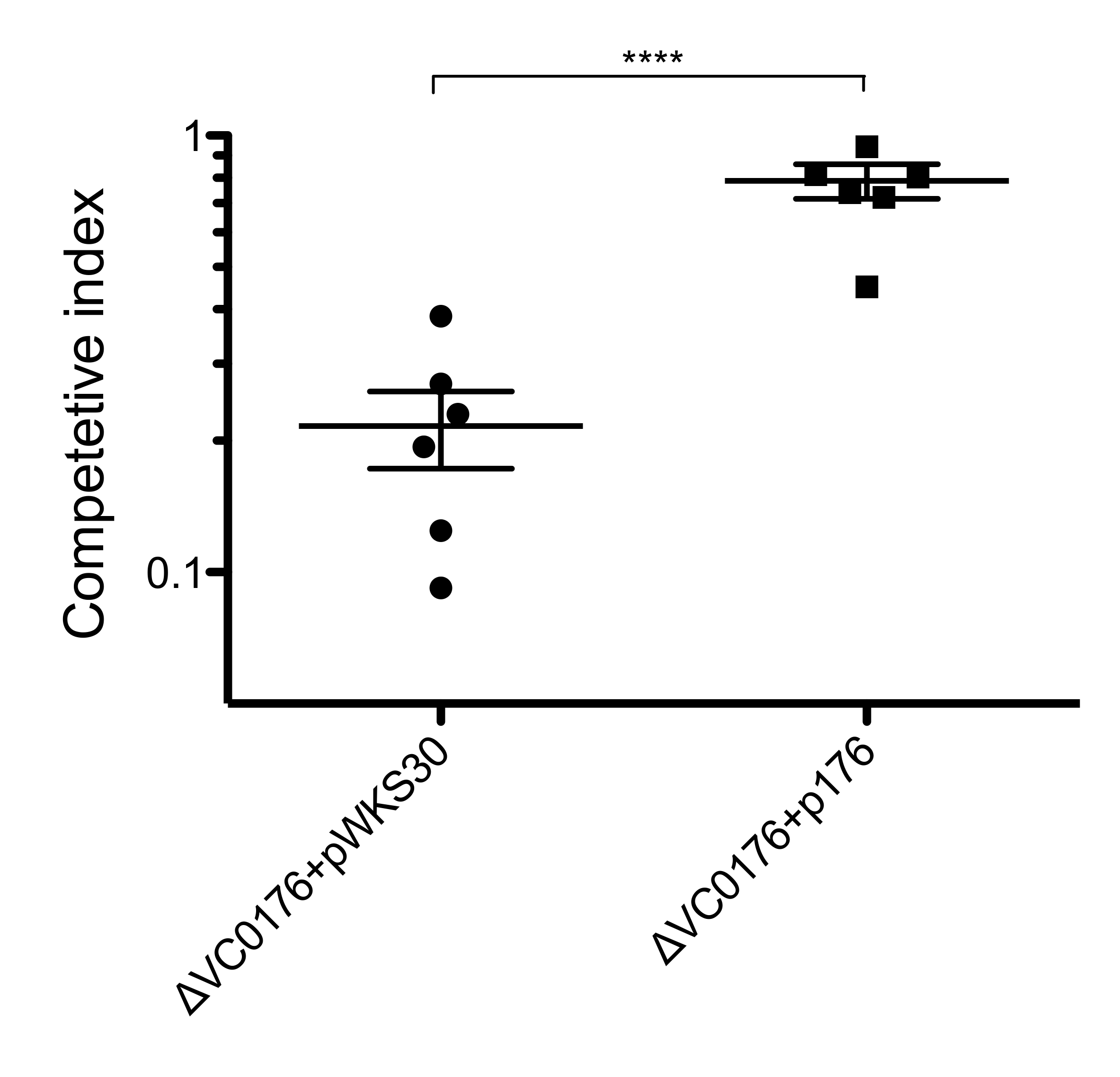

Supplement: S7 Fig — Results of infant mouse colonization assays of each indicated strain competed against the wild type strain carrying empty vector. pWKS30 is the empty vector. p176 is the complementing plasmid expressing VC1076 from its native promoter. ΔVC0176+pWKS30 showed a defect in infant mouse colonization compared to the complemented strain ΔVC0176+p176. ****p < 0.0001, unpaired two-tailed Student’s t test. (TIF) [file ppat.1005570.s007.tif]

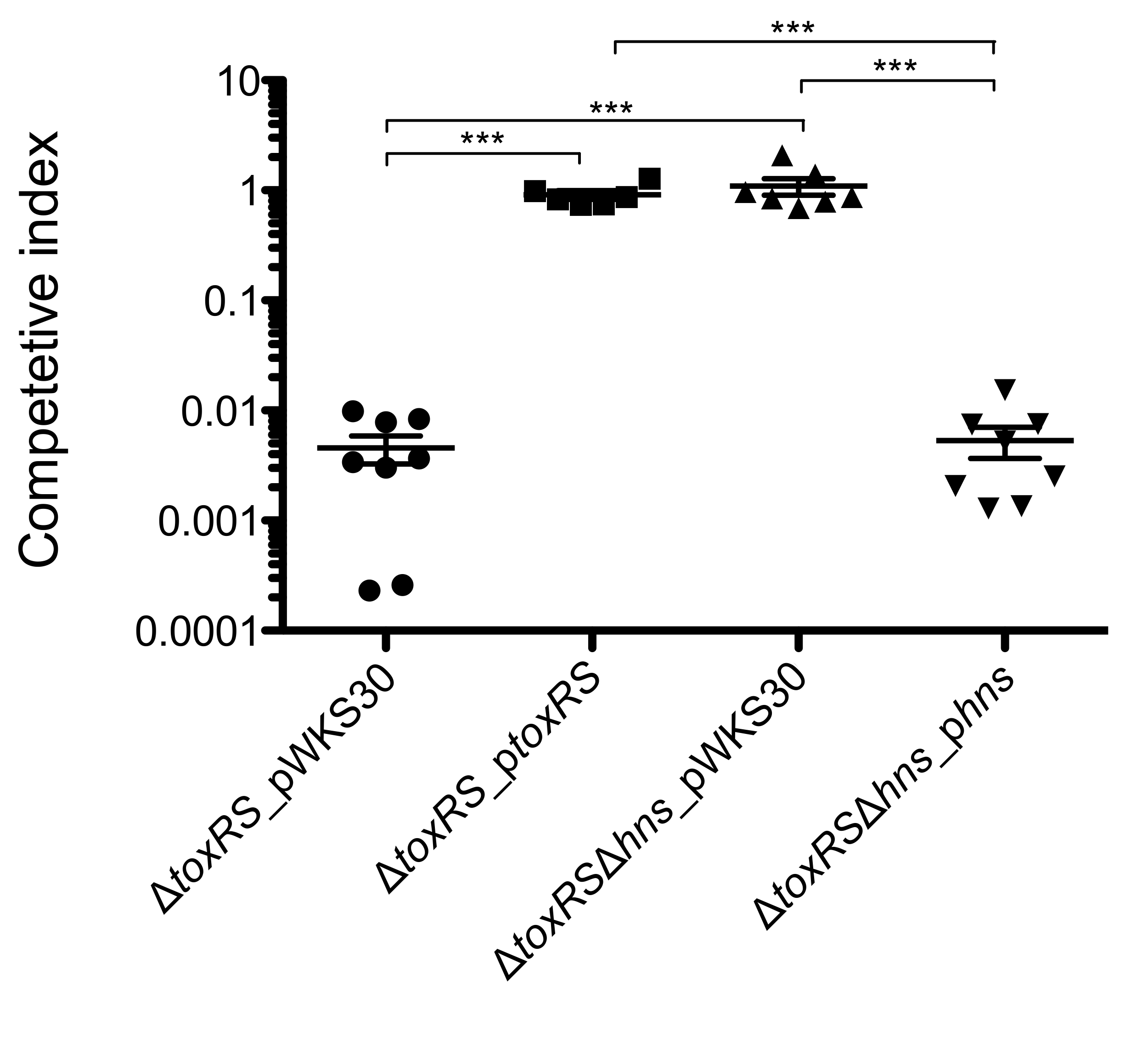

Supplement: S8 Fig — Results of infant mouse colonization assays of each indicated strain competed against the wild type strain carrying empty vector. pWKS30 is the empty vector. ptoxRS and phns are complementing plasmids expressing toxRS and hns respectively under their native promoters. ΔtoxRS+pWKS30 showed a defect in infant mouse colonization compared to the complemented strain ΔtoxRS+ptoxRS, as well as ΔtoxRSΔhns+pWKS30. ΔtoxRSΔhns+phns showed a defect in colonization of the infant mouse intestine compared to ΔtoxRSΔhns+pWKS30, as well as ΔtoxRS+ptoxRS. Statistical significance was determined by One-Way ANOVA analysis followed by a Tukey’s multiple comparison post-test, ***p < 0.001. (TIF) [file ppat.1005570.s008.tif]

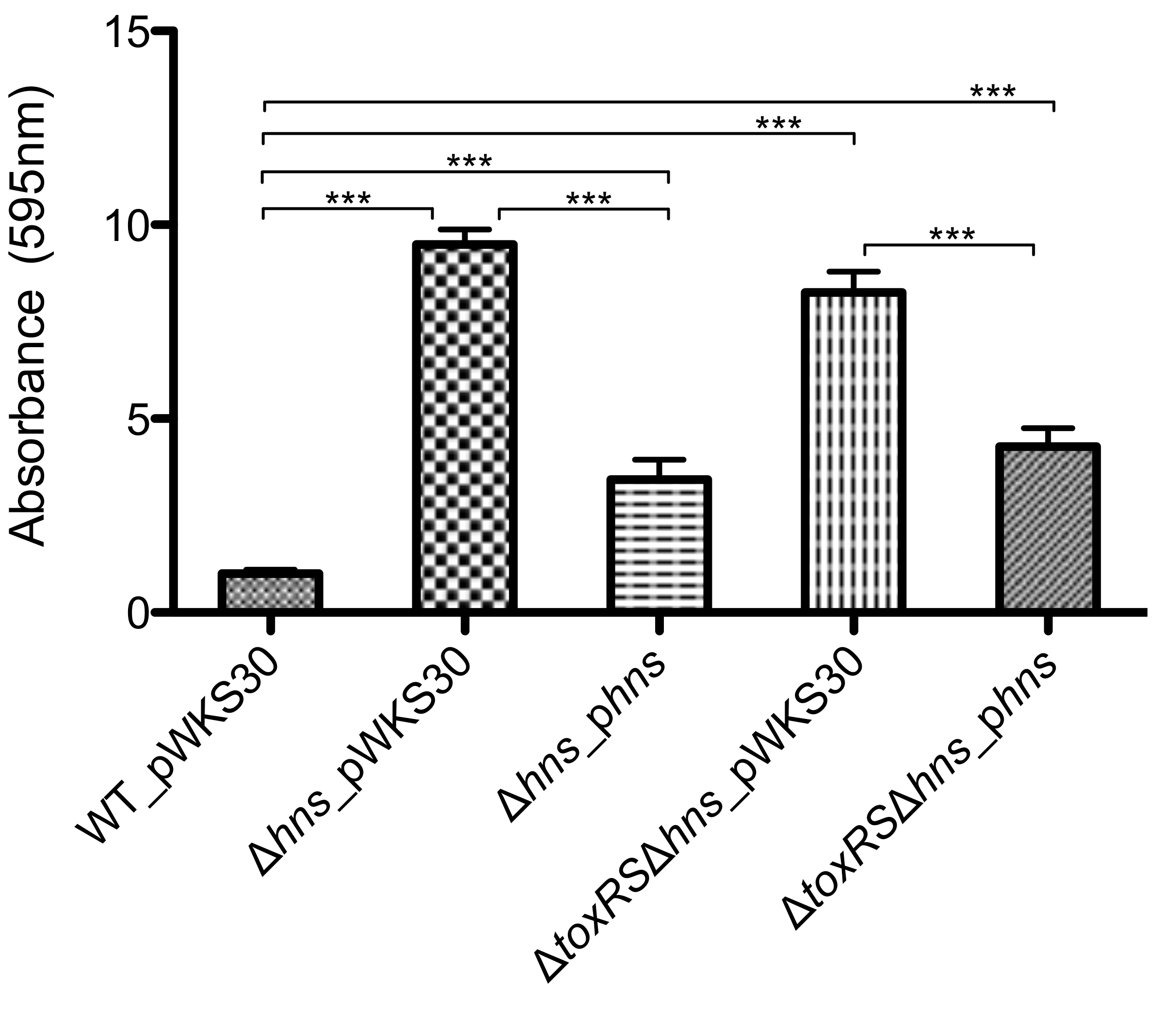

Supplement: S9 Fig — Biofilm assays were performed as described in the methods. All biofilm measurements were normalized to the wild type strain carrying the empty vector pWKS30, which was set to 1. phns is the plasmid encoding hns expressed from its native promoter. Each mutant showed increased biofilm production compared to the WT+pWKS30 strain. Δhns+phns showed a defect in biofilm formation compared to Δhns+pWKS30. ΔtoxRSΔhns+phns showed a defect in biofilm formation compared to ΔtoxRSΔhns+pWKS30. Statistical significance was determined by One-Way ANOVA analysis followed by a Tukey’s multiple comparison post-test, ***p < 0.001. Standard error of the mean (SEM) is shown. (TIF) [file ppat.1005570.s009.tif]
