# Supplementary material for: ToxR Antagonizes H-NS Regulation of Horizontally Acquired Genes to Drive Host Colonization
Source: PLoS Pathog. 2016 Apr 12;12(4):e1005570. doi: 10.1371/journal.ppat.1005570 (PMC4829181; doi:10.1371/journal.ppat.1005570)
Supplement: S1 Table — (DOCX) [file ppat.1005570.s010.docx]

| Associated Gene(s) | 5’ genome coordinate | 3’ genome coordinate |
| --- | --- | --- |
| *ryhB* | 101613 | 102938 |
| VC0176-VC0178 | 174870 | 179289 |
| VC0182-VC0183 | 184362 | 186750 |
| VC0260 (*galE*) | 266024 | 268478 |
| VC0269 (*manA*) | 274420 | 275209 |
| VC0280 (*cadB*) | 284937 | 285956 |
| VC0423 | 454028 | 455150 |
| VC0490 | 524510 | 526282 |
| VC0493 | 527218 | 528473 |
| VC0501 | 533740 | 534775 |
| VC0633 (*ompU*) | 675753 | 676688 |
| VC0824 | 885809 | 886959 |
| VC0825 (*tcpI*) | 887063 | 888602 |
| VC0838 (*toxT*) | 899355 | 900173 |
| VC0844 (*acfA*) –VC0845 (*acfD*) | 904894 | 906799 |
| VC0880 | 940748 | 941510 |
| VC0934 (*vpsL*) | 999096 | 1000387 |
| VC0972 | 1036625 | 1038223 |
| VC0988 (*tppB*) | 1053668 | 1054491 |
| VC1145 | 1216742 | 1217615 |
| VC1197 | 1269678 | 1270352 |
| VC1330 | 1413054 | 1414298 |
| VC1398 (*cheY*) | 1489540 | 1490309 |
| VC1599 | 1712160 | 1713491 |
| VC1613 | 1727175 | 1728060 |
| VC1649 | 1776112 | 1776761 |
| VC1762 | 1902193 | 1903284 |
| VC1773 | 1923340 | 1924138 |
| VC1800 | 1947116 | 1947916 |
| VC1839 | 1980022 | 1981588 |
| VC1854 (*ompT*) | 1993285 | 1994750 |
| VC1856 | 1995467 | 1996457 |
| VC2013 | 2168041 | 2168957 |
| VC2485 (*leuO*) | 2666758 | 2668014 |
| VC2697 | 2863843 | 2864948 |
|  |  |  |

Table S1. Coordinates of raw ToxR ChIP-seq peaks and associated genes*.

*The coordinates listed here are the positions of the maximum boundary points encompassing raw ToxR peaks identified in our sequence analysis and have not been deconvoluted.
