# Supplementary material for: ToxR Antagonizes H-NS Regulation of Horizontally Acquired Genes to Drive Host Colonization
Source: PLoS Pathog. 2016 Apr 12;12(4):e1005570. doi: 10.1371/journal.ppat.1005570 (PMC4829181; doi:10.1371/journal.ppat.1005570)
Supplement: S4 Table — (DOCX) [file ppat.1005570.s013.docx]

Table S4. Strain and plasmid list.

| **Strain/Plasmid** | **Description** | **Refernce/Source** |
| --- | --- | --- |
| **Strains** |  |  |
| C6706 | Wild-type V. cholerae El Tor biotype, Sm^R^ | Lab stock |
| MIK01 | C6706 ∆*toxRS* | This Study |
| MIK02 | C6706 ∆*tcpPH* | This Study |
| MIK03 | C6706 ∆VC1599 | This Study |
| MIK04 | C6706 ∆VCA0536 | This Study |
| MIK05 | C6706 wild-type carrying pBAD18Cm | This Study |
| MIK06 | C6706 wild-type carrying pWKS30 | This Study |
| MIK07 | C6706 ∆*toxRS* carrying pWKS30 | This Study |
| MIK08 | C6706 ∆VC1599 carrying pWKS30 | This Study |
| MIK09 | C6706 ∆*toxRS* carrying pMK01 | This Study |
| MIK10 | C6706 ∆*tcpPH* carrying pMK02 | This Study |
| MIK11 | C6706 ∆VC1599 carrying pMK03 | This Study |
| MIK12 | C6706 ∆*toxRS* carrying pBAD18 | This Study |
| MIK13 | C6706 ∆*tcpPH* carrying pBAD18Cm | This Study |
| MIK14 | C6706 ∆*toxRS* carrying pMK05 | This Study |
| MIK15 | C6706 ∆*tcpPH* carrying pMK06 | This Study |
| MIK16 | C6706 wild-type carrying pMK05 | This Study |
| MIK17 | C6706 wild-type carrying pMK06 | This Study |
| MIK18 | C6706 ∆*ryhB::* kan^R^ carrying pWKS30 | This Study |
| MIK19 | C6706 ∆*ryhB::* kan^R^ carrying pMK07 | This Study |
| MIK20 | C6706 ∆*toxRS*_∆*hns* | This Study |
| ARC01 | C6706 ∆*hns* | This Study |
| MIK21 | C6706 ∆*ryhB::* kan^R^ | [46] |
| MIK22 | C6706 ∆VC0176 | This Study |
| MIK23 | C6706 WT carrying pBAD18Cm +*toxR*-3XV5 C-term (C6706 *toxR*) | This Study |
| MIK24 | C6706 WT pBAD18Cm + *tcpP*-3XV5 C-term (C6706 *tcpP*) | This Study |
|  | *E. coli* SM10 λpir | Lab stock |
|  | *E. coli* C2987 | NEB |
|  | *E. coli* Pir1 | Invitrogen |
| ARC02 | C6706 WT H-NS 1XV5 tag | This Study |
| MIK25 | C6706 WT H-NS 1XV5 tag carrying pBAD18Kn | This Study |
| MIK26 | C6706 WT H-NS 1XV5 tag carrying pMK06 | This Study |
| MIK27 | C6706 ∆VC0176 carrying pWKS30 | This Study |
| MIK28 | C6706 ∆VC0176 carrying pMIK08 | This Study |
| MIK29 | C6706 ∆*hns* carrying pWKS30 | This Study |
| MIK30 | C6706 ∆*hns* carrying pMIK09 | This Study |
| MIK31 | C6706 ∆*vpsL* | This Study |
| MIK32 | C6706 ∆*vpsL* carrying pWKS30 | This Study |
| MIK33 | C6706 ∆*vpsL* carrying pMIK10 | This Study |
| MIK34 | C6706 ∆*toxRS*_∆*hns* carrying pWKS30 | This Study |
| MIK35 | C6706 ∆*toxRS*_∆*hns* carrying pMIK09 | This Study |
|  |  |  |
| **Plasmids** |  |  |
| pBAD18Cm | cm^R^ | [91] |
| pBAD18Kn | kan^R^ | [91] |
| pWM91 | amp^R^ | [89] |
| pSSK10 | cm^R^/kan^R^ | [90] |
| pWKS30 | amp^R^ | [92] |
| pMIK01 | pWKS30 carrying *toxRS* with native promoter (p*toxRS*) | This Study |
| pMIK02 | pWKS30 carrying VC1599 with native promoter (pVC1599) | This Study |
| pMIK03 | pBAD18Cm carrying *toxR*-3XV5 C-term (C6706 *toxR*) | This Study |
| pMIK04 | pBAD18Cm carrying *tcpP*-3XV5 C-term (C6706 *tcpP*) | This Study |
| pMIK05 | pWKS30 carrying sRNA *ryhB* with native promoter (p*ryhB*) | This Study |
| pMIK06 | pBAD18Kn carrying *toxRS* (p*toxRS*) | This Study |
| pMIK07 | pBAD18Kn carrying *tcpPH* (p*tcpPH*) | This Study |
| pMIK08 | pWKS30 carrying VC0176 with native promoter (pVC0176) | This Study |
| pMIK09 | pWKS30 carrying *hns* with native promoter (p*hns*) | This Study |
| pMIK10 | pWKS30 carrying *vpsL* with native promoter (p*vpsL*) | This Study |
