# Supplementary material for: ToxR Antagonizes H-NS Regulation of Horizontally Acquired Genes to Drive Host Colonization
Source: PLoS Pathog. 2016 Apr 12;12(4):e1005570. doi: 10.1371/journal.ppat.1005570 (PMC4829181; doi:10.1371/journal.ppat.1005570)
Supplement: S5 Table — (DOCX) [file ppat.1005570.s014.docx]

Table S5. Primer list

| **Application** | **Primer** | **Sequence (5’ to 3’)** |
| --- | --- | --- |
| ChIP Primers | toxTCh5 | GGATGGCTCTCTGCGTTTAT |
|  | toxTCh3 | TTTCCCAATCATTGCGTTCT |
|  | VC0490Ch5 | CATTTAGTGGTACATCGGGTTC |
|  | VC0490Ch3 | GCTAAACGCAAGGCTATCTTACA |
|  | VC0633Ch5 | GCCACGTCAATCGAAATAGG |
|  | VC0633Ch3 | GCGGAACTTTGGGAGTAAAA |
|  | VC0824Ch5 | TGGCCCTATATGTCCGCTTA |
|  | VC0824Ch3 | TTTAGTGTCACCGCTATTATACAAAC |
|  | VC0825_VC0826Ch5 | CAAACGTAAGGGGCAAAATG |
|  | VC0825_VC0826Ch3 | TGCATAATAACCAAGCCATTCA |
|  | VC0844_VC0845Ch5 | TCCTATTTTTACCTGTGTTTCACATT |
|  |  |  |
|  | VC0844_VC0845Ch3 | CAATACGAATCTTCATCTTAAGCCTAT |
|  | VC1762Ch5 | AATCCTCTCGAGCTCAGCAA |
|  | VC1762Ch3 | TGGAATGTCTGGCAAAATGA |
|  | VC1773Ch5 | TAGAGCCATTCCAAGCCAAC |
|  | VC1773Ch3 | CACATGAAGGGCTGCCTAAG |
|  | VC1800Ch5 | TCCAATGCGATCAACTTCTG |
|  | VC1800Ch3 | CATTAAGGGCGCAACTTTTC |
|  | VC0176Ch5 | TGCTTCTTTGAGACGCAGTG |
|  | VC0176Ch3 | TGAGCCATAAGTGTCATTCCA |
|  | VC0178Ch5 | GCAATGGTCCAGGGTATGAG |
|  | VC0178Ch3 | GGTGGATTTGGCATGTAACC |
|  | VC0182Ch5 | CCCATTGATAGCGTTTGTCC |
|  | VC0182Ch3 | CCCCAGCCAACTGTATGAGT |
|  | VC0183Ch5 | TGGCCATAGAGGGTGTCTTC |
|  | VC0183Ch3 | TTCACCATCCATCCCAAAGT |
|  | VC0493Ch5 | GGATTTAGCCGATGTGATGG |
|  | VC0493Ch3 | GAGGTCGTTGGTTGAGGTGT |
|  | VC2485Ch5 | AACATTTGCATGCCTCACAG |
|  | VC2485Ch3 | CCAAATATCTGGGCGGTAAA |
|  | VC1599Ch5 | TCGTTTGGCTTTTCTGTCATC |
|  | VC1599Ch3 | ATCCATGCACTCACCCTCTC |
|  | VC0934Ch5 | TGGTTGTTTTCAATTGGTTTCA |
|  | VC0934Ch3 | AGACGCTCCTAACCGATGTAA |
|  | VCA0536Ch5 | TCCACTGTAACACGGCGTAA |
|  | VCA0536Ch3 | ATTCGCAATTAACCCAAACG |
|  | ryhBRT5 | TGAAGGTTCACCAGGTT |
|  | ryhBRT3 | CAAACGAGGTCAAAGCCAAT |
| Relative Gene Expression | VC16SRT5 | CAGCCACACTGGAACTGAGA |
|  | VC16SRT3 | TTAGCCGGTGCTTCTTCTGT |
|  | icdRT5 | CGGGACAAGACACCATTTCTG |
|  | icdRT3 | AGAGAGCATTTTCGCCGAAG |
|  | VCA0536RT5 | AACCTTGCGCAGTGTTTTTC |
|  | VCA0536RT3 | GCGAAGGTTTCAGAATGGAA |
|  | VC1854RT5 | TGGGTTAAAGCTGGTTACGG |
|  | VC1854RT3 | CACAGCCAAATCACCAAATG |
|  | VC1599RT5 | GCCTTTAATGATGGGGGTTT |
|  | VC1599RT3 | GGAGCACTGACCAAGAGAGG |
|  | ToxTRT5 | AGCTGTCCTTTCTGAAGTGG |
|  | ToxTRT3 | TTCGAGAAGAACCCTGAAAAA |
|  | VC0934RT5 | AAATTTTGGATGCGTTTTCG |
|  | VC0934RT3 | ATAGCGACGGCCAATAACAC |
|  | VC0176RT5 | CAGAAAGCCTTGGGAGTACG |
|  | VC0176RT3 | TTCTCTTCAGGGGACATTCG |
|  | VC0178RT5 | ATTTCGTGCGCTTGGATAAC |
|  | VC0178RT3 | TACTGAGCGAGCTTGCAGAA |
|  | VC0493RT5 | GCTGGTTTCGACACTTCTCC |
|  | VC0493RT3 | TTGCCTTATGGTTCGAGCTT |
|  |  |  |
|  | toxRS_pWKS30_For | ATAGAGCTCGTTGGAGTACAAAGAGTGAATCATCAGGTG |
|  | toxRS_pWKS30_Rev | ATAGGTACCTTAAGAATTACTGAACAGTACGGTAGAACCATG |
|  | VC1599_pWKS30_For | ATAGTCGACGAGAACGAGGTGGATATTAGCGGC |
|  | VC1599_pWKS30_Rev | ATAGGTACCTCACACGTTCTCGGTTTGTTGATTAACCATAGTAGG |
|  | tcpPH_pWKS30_For | ATAGTCGACCTAAGTTTAAATGGTTATCACGGAGTACTTCGTG |
|  | tcpPH_pWKS30_Rev | ATAGGTACCCTAAAAATCGCTTTGACAGGAAAACCACATTTTTTCAG |
|  | ryhB_pWKS30_For | ATAGTCGACGGGCATGGTGTCGCGCAGCGCTTACTT |
|  | ryhB_pWKS30_Rev | ATAGGTACCCCCACTTATCCAACCCTACATTCTGTGG |
|  | VC1130_pWKS30_For | ATAGGATCCCAAGTGCCACAGCTCACAAATCAATC |
|  | VC1130_pWKS30_Rev | ATAGTCGACTTACAGAGCGAATTCTTCCAGAGATTTACC |
|  | VC0176_pWKS30_For | ATAGGATCCCGCTTCATCAGGATCAATGAGTGAGTC |
|  | VC0176_pWKS30_Rev | ATAGTCGACCTATTTCTTACTAATTAAATCTTTTAGAAGG |
|  | VC0934_pWKS30_For | ATAGGATCCCATGGTGGTTAATAAGTGAGTCTCAAGG |
|  | VC0934_pWKS30_Rev | ATAGTCGACTTAATACGCGTTTTTTCCAACAAATCCTTTG |
|  | VC1130CDU5 | ATAGGGCCCAGTACACGGCGAGTCACAATAGCCAAACTC |
|  | VC1130CDU3 | CGGGAGCCTTTATCATTTTAGTTTCTGGCAAAATTACAGCGACATTACCATTTCCTGTTCACAATTTGG |
|  | VC1130CDD5 | CCAAATTGTGAACAGGAAATGGTAATGTCGCTGTAATTTTGCCAGAAACTAAAATGATAAAGGCTCCCG |
|  | VC1130CDD3 | ATACTCGAGCTCAACTATCTGCAAGGTGTAGATGGC |
|  | VC1130XV5U5 | ATAGGGCCCTGGATACTTTTCGTAACCGCTCCTCTACGG |
|  | VC11301XV5U3 | TTAAGTAGAATCTAAACCAAGGAGAGGATTAGGAATAGGTTTACCAGCAGCCAGAGCGAATTCTTCCAGAGATTTACCTTC |
|  | VC11301XV5D5 | GGTAAACCTATTCCTAATCCTCTCCTTGGTTTAGATTCTACTTAATTTTGCCAGAAACTAAAATGATAAAGGCTCCCG |
|  | VC1130XVCD3 | ATACTCGAGCCAATGCCGGAAGCAACAATGCGTGCC |
|  | VC1130CDcon5 | CTCTTTGACAAACACGCTACCCACTTGTGTGGC |
|  | VC1130CDcon3 | CGTTTGATTACCAATGAATATGCAGGTGGAACC |
|  | VCA0536U5 | ATACTCGAGCACAAGGCAGTGGCGCAGCAGGC |
|  | VCA0536U3 | GCAAGATTGACAGAATGAACGTTTCGCCAATAACATGTAAATACTCGTCAAGATAGGAGGCATCAGCGG |
|  | VCA0536D5 | CCGCTGATGCCTCCTATCTTGACGAGTATTTACATGTTATTGGCGAAACGTTCATTCTGTCAATCTTGC |
|  | VCA0536D3 | ATAGGGCCCCATCCTTGCGAATACGCCCCCATCG |
|  | VCA0536SP5 | GAGCGTACTACAGGGAAGAAGCGACC |
|  | VCA0536SP3 | CAAGAAGTCACCGTTTCCTGCTAGTGAC |
|  | ryhBprobe1 | TCGCTACCGCATATCGATGACTCGATAAAGGGTGAC |
|  | VC5Sprobe1 | CCCACACTACCATCGACGCTGTTTCGTTTCACTTCTGAGTTC |
|  | toxRpBAD5’ | ATAGGTACCAGGAGGAACGATGTTCGGATTAGGACACAACTCAAAGAG |
|  | toxR3XV53 | ATAGTCGACCTCACACACTTTGATGGCATCGTTAGG |
|  | tcpPpBAD5 | ATAGGTACCAGGAGGAAACGATGGGTTATGTCCGCGTGATTTATCAATTTCC |
|  | tcpP3XV53 | ATAGTCGACATTTTTTGTGCATTCTAATGTCTTCTGTTCATAATTCACGG |
|  | tcpPHCDU5 xhoI | ATACTCGAGCGTTGAGTTCTTCGACTGCTTTATCGCG |
|  | tcpPHCDU3 | CTATCTAGGCGGCTCATGATAAGACCCTAGGGAAATTGATAAATCACGCGGACATACCC |
|  | tcpPHCDD5 | GGGTATGTCCGCGTGATTTATCAATTTCCCTAGGGTCTTATCATGAGCCGCCTAGATAG |
|  | tcpPHCDD3 apaI | ATAGGGCCCCAGCGTAACAACACCCGCTGAGACC |
|  | tcpPHSP5 | GCGACACTGCCGACAATGTCGTTGC |
|  | tcpPHSP3 | GTGCTTGGGTCAAGCCACCGACTG |
|  | VC1599U53 apaI | ATAGGGCCCCATTTGCAAGATTTGATCACCCACCACCCC |
|  | VC1599U32 | CGCCAGCAATCCCAGCGTTAATTAAACTCACATGCACTCACCCTCTCCGG |
|  | VC1599D52 | CCGGAGAGGGTGAGTGCATGTGAGTTTAATTAACGCTGGGATTGCTGGCG |
|  | VC1599D3 | ATAGGGCCCCCGAGAAGTATTGGGGAGAATCGCTCTCC |
|  | VC1599SP5 | GCTTGCCAGTGAGCCAAGAAGAGTTTG |
|  | VC1599SP3 | CCAAAATGGAGAGTGGCGTGGCCTATAC |
|  | ryhBCDSP5 | GGTGTCGCGCAGCGCTTACTT |
|  | ryhBCDSP3 | TTTAAGTTAATGTTTTTAAAC |
|  | ToxRSCDU53 | ATAGGATCCTTCAGCCGTACCCGATTTAGCAATCGTACC |
|  | ToxRSCDU3 | GTCCTATTCTACGCTCAGTCAGGTTAAGAGTGTCCTAATCCGAACATCTAATGTCCCAG |
|  | ToxRSCDD5 | CTGGGACATTAGATGTTCGGATTAGGACACTCTTAACCTGACTGAGCGTAGAATAGGAC |
|  | ToxRSCDD32 | ATAGGGCCCGTTTTCCTATGATGAGTGACTCAGCATGGG |
|  | ToxRScon5 | CTTTATTGATAGTTTCAACCGTGTACTCGC |
|  | ToxRScon3 | CTTTATCTGCGACCGAACAAGCACTTGCGC |
|  | VC0176CDU25 | ATACTCGAGAACCTTTCCCAGTAGACACGCAGAACG |
|  | VC0176CDU3 | CACTAAATATAGTGTCTCTATCTATTTCTTACTGAATGACACTAATGGCTCATAATCTTGAAG |
|  | VC0176CDD5 | CTTCAAGATTATGAGCCATTAGTGTCATTCAGTAAGAAATAGATAGAGACACTATATTTAGTG |
|  | VC0176CDD23 | ATAGGGCCCCCTGAAATTATGAAACTTATTTCTATACTCTCAGG |
|  | VC0176con5 | TAAAAGCACTCTCAAAACCTCAAGCAC |
|  | VC0176con3 | TAATGCCTTTACACCAGAGCCAATAGC |
|  | VC0934U5 | ATACTCGAGCGCATGCGTCAACGGCGAGTTG |
|  | VC0934U3 | GTGCCCTGTCTTAATACGCGTTTTTTCCGCTTTTTTCCTTCATCACTAGACGCTCC |
|  | VC0934D5 | GGAGCGTCTAGTGATGAAGGAAAAAAGCGGAAAAAACGCGTATTAAGACAGGGCAC |
|  | VC0934D3 | ATA GGGCCC CTTTGCTGCTGGCTCCCGGTG |
|  | VC0934SP5 | GTTCGGTGATGCAGTACCACCACC |
|  | VC0934SP3 | GCGGCTTCAGTCGTCCATTGGG |
